# Applications of microalgal biofilms for wastewater treatment and bioenergy production

**DOI:** 10.1186/s13068-017-0798-9

**Published:** 2017-05-10

**Authors:** Ana F. Miranda, Narasimhan Ramkumar, Constandino Andriotis, Thorben Höltkemeier, Aneela Yasmin, Simone Rochfort, Donald Wlodkowic, Paul Morrison, Felicity Roddick, German Spangenberg, Banwari Lal, Sanjukta Subudhi, Aidyn Mouradov

**Affiliations:** 1 School of Sciences, RMIT University, Bundoora, VIC Australia; 2 The Energy and Resources Institute, New Delhi, 110 003 India; 3 Technical University of Braunschweig, Brunswick, Germany; 4 Sindh Agriculture University, Tandojam, Pakistan; 5 AgriBio, Centre for AgriBioscience, La Trobe University, Bundoora, VIC 3083 Australia; 6 School of Engineering, RMIT University, Bundoora, VIC Australia

**Keywords:** Biofilms, Bio-hydrogen, Biofuel, Cyanobacteria, Microalgae, Wastewater treatment

## Abstract

**Background:**

Microalgae have shown clear advantages for the production of biofuels compared with energy crops. Apart from their high growth rates and substantial lipid/triacylglycerol yields, microalgae can grow in wastewaters (animal, municipal and mining wastewaters) efficiently removing their primary nutrients (C, N, and P), heavy metals and micropollutants, and they do not compete with crops for arable lands. However, fundamental barriers to the industrial application of microalgae for biofuel production still include high costs of removing the algae from the water and the water from the algae which can account for up to 30–40% of the total cost of biodiesel production. Algal biofilms are becoming increasingly popular as a strategy for the concentration of microalgae, making harvesting/dewatering easier and cheaper.

**Results:**

We have isolated and characterized a number of natural microalgal biofilms from freshwater, saline lakes and marine habitats. Structurally, these biofilms represent complex consortia of unicellular and multicellular, photosynthetic and heterotrophic inhabitants, such as cyanobacteria, microalgae, diatoms, bacteria, and fungi. Biofilm #52 was used as feedstock for bioenergy production. Dark fermentation of its biomass by *Enterobacter cloacae* DT-1 led to the production of 2.4 mol of H_2_/mol of reduced sugar. The levels and compositions of saturated, monosaturated and polyunsaturated fatty acids in Biofilm #52 were target-wise modified through the promotion of the growth of selected individual photosynthetic inhabitants. Photosynthetic components isolated from different biofilms were used for tailoring of novel biofilms designed for (i) treatment of specific types of wastewaters, such as reverse osmosis concentrate, (ii) compositions of total fatty acids with a new degree of unsaturation and (iii) bio-flocculation and concentration of commercial microalgal cells. Treatment of different types of wastewaters with biofilms showed a reduction in the concentrations of key nutrients, such as phosphates, ammonia, nitrates, selenium and heavy metals.

**Conclusions:**

This multidisciplinary study showed the new potential of natural biofilms, their individual photosynthetic inhabitants and assembled new algal/cyanobacterial biofilms as the next generation of bioenergy feedstocks which can grow using wastewaters as a cheap source of key nutrients.

**Electronic supplementary material:**

The online version of this article (doi:10.1186/s13068-017-0798-9) contains supplementary material, which is available to authorized users.

## Background

Extensive use of arable lands and high consumption of freshwater by energy crops have triggered an intensive search for the next generation of bioenergy feedstocks which will meet key selection criteria: (i) high growth rates/biomass production; (ii) high content of bioenergy-producing molecules; (iii) high harvesting index and short rotation period; (iv) ability to grow on marginal lands and lack of competition with agricultural crops for arable lands; (v) low freshwater usage; (vi) low costs for growth and harvest; and (vii) production of high-value co-products [1]. The use of wastewater as a source of key nutrients would significantly improve the economics of biofuel production and reduce its energy requirements. This has shifted attention from the application of terrestrial energy crops towards the use of microalgae and aquatic plants. Microalgae have shown clear advantages for the production of biofuels compared with energy crops. Apart from their high-growth rates and substantial lipid/triacylglycerol (TAG) yields, microalgae can grow in wastewater efficiently removing the primary nutrients, heavy metals, and micropollutants. And, microalgae do not compete with crops for arable land [2–4]. However, some fundamental barriers to the industrial application of microalgae for biofuel production still exist and include high costs for their growth, harvesting and high freshwater requirements. The main techniques used for harvesting and concentration of microalgal cells (centrifugation, filtration, flocculation, gravity sedimentation and flotation) are still not economically viable for the large-scale microalgal industry [5–13]. Bio-flocculation methods are becoming increasingly popular because of their high efficiency and low energy input [9, 14, 15]. Fungal-assisted bio-flocculation as a new strategy for attachment and concentration of microalgal cells within fungal filaments, via hydrogen bonds, electrostatic interactions and/or using a matrix of extracellular polymeric substance (EPS) secreted by fungal and algal cells, started attracting attention since it was shown to be highly efficient for the concentration of microalgal cells, does not require added chemicals and has a low energy input requirement [6, 16–20]. Moreover, co-cultivation of microalgae and a filamentous fungus showed an additive effect on total biomass production, lipid yield, and wastewater treatment efficiency. Application of alternative sources of carbon from lignocellulosic wastes, nitrogen, and phosphorus from wastewaters for fungal and algal growth improves the economics of biofuel production [19, 20]. This strategy, however, is limited because of environmental concerns over potential contamination of treated water with fungal spores as a result of large-scale fungal production. Moreover, the addition of carbon sources such as glucose for heterotrophic fungal growth under non-sterile conditions will trigger growth of microbial populations, which will significantly limit the application of this technology under environmental, non-axenic conditions. This concern has triggered a search for alternative environmentally friendly and low-cost strategies for algal bio-flocculation, where all components can create structures which are stable under the natural conditions, and can cumulatively contribute to the production of the total biomass and added value products.

In natural ecosystems, algal biofilms represent three-dimensional, multilayered and multispecies structures which involve consortia of heterotrophic and photoautotrophic prokaryotic and eukaryotic organisms [21–27]. Photosynthetic organisms include filamentous and unicellular macro- and microalgae, and cyanobacterial species. These organisms are characterized by their typical pigment content, which is used to capture solar energy and to protect the cells from radiation [28]. Heterotrophic organisms can include protozoa, flagellates, bacterial and fungal cells. In this highly heterogeneous structure, different species usually colonize different zones within the biofilm structure that are most suitable for their growth [21]. Heterotrophic and photoautotrophic organisms in this complex and highly productive ecosystems can be held together by a matrix of EPS, the functions of which include adhesion, aggregation, retention of water and nutrients, diffusion barrier for toxins and heavy metals, cell motility, protection barriers against grazers and harmful chemicals or environmental conditions [24, 29–31]. EPS, which can account for 90% of dry biofilm mass, are typically composed of the high molecular weight heteropolysaccharides containing linear or branched repeating units comprising 2–10 monosaccharides, such as hexoses, pentoses, glucose, mannose, arabinose, uronic acids, and deoxy-sugars. A low contamination of intracellular polymers and proteins in EPS suggests low rates of cell rupture within biofilms [24].

Wastewater treatment is one of the favourable applications of algal biofilm systems because they offer a simple, energy-efficient technology for absorption of the key nutrients, nitrogen and phosphorus followed by easy and robust separation of the algal biomass from the bulk of the wastewater [32–37]. However, in spite of the obvious advantages of low-cost nutrient removal and low energy biomass production of biofilm-based technology, widespread application of algal biofilm-based treatment of municipal, industrial, and agricultural waste streams has been limited so far [38].

Reverse osmosis (RO) technology is being used worldwide for full-scale municipal wastewater reclamation to produce high-quality recycled water to meet the increasing water supply demand [39]. However, this technology generates highly saline RO concentrate (ROC) streams, which contain almost all of the contaminants and nutrients derived from the secondary effluent at elevated levels (4–6 times higher) [40]. With commercial scale capacities up to 3 million m^3^/day of clean water, generation of ROC at large scale creates a significant ecological problem. Consequently, there is a growing need to explore cost-effective treatment options for the ROC for reducing its environmental and health risks on disposal or reuse.

Hydrogen is one of the cleanest forms of renewable energy which can be generated from different alternate sources, through thermochemical, electrochemical and biological processes [41]. Bio-hydrogen production through dark fermentation route has attracted substantial attention from researchers as this process can utilise organic wastes as substrate [42–45], and is not energy intensive. Hence, this process can lead to light-independent biodegradation of biomass with simultaneous energy recovery from waste.

In this work, we explored the potentials of natural biofilms, their photosynthetic inhabitants and newly assembled algal/cyanobacterial biofilms as the next generation of bioenergy feedstocks which can grow using wastewaters as a cheap source of key nutrients. To our knowledge, for the first time composition of algal/cyanobacterial biofilms was tailored for treatment of specific types of wastewaters and for the production of lipids with specific compositions of fatty acids which meet key biodiesel characteristics, such as iodine number, cetane number, density, pour point, viscosity and others. We also believe this is the first report on the use of algal/cyanobacterial biofilms as a feedstock for bio-hydrogen production.

## Results

### Characterization of natural microalgal biofilms

#### Characterization of Biofilm #52

We have isolated and characterized a number of natural microalgal biofilms from the saline lakes and marine habitats around Melbourne, Victoria, Australia. Some of them are shown in Additional file 1: Figure S1. Structurally, these biofilms represent complex consortia of unicellular and multicellular (filamentous) photosynthetic and heterotrophic components, such as cyanobacteria, green-algae, diatoms, bacteria, and fungi. The photosynthetic components were identified by the characteristic red fluorescence of their chlorophyll molecules. Typically, the photosynthetic inhabitants of the biofilms comprise 1–2 filamentous cyanobacteria, 1–3 green microalgae, and 2–4 diatom species. Within the biofilms, the individual photosynthetic inhabitants normally colonize different parts of the biofilm’s surface, not always mixing with each other (Additional file 2: Figure S2). Growing from the small amount of seed culture on artificial seawater growth media (F2) the biofilms easily created mats which were both attached to the walls and freely floating in the medium (Additional file 3: Figure S3A). The biofilms and their isolated photosynthetic components were also efficiently grown on solid, agar-containing medium (Additional file 3: Figure S3B).

**Fig. 1:**
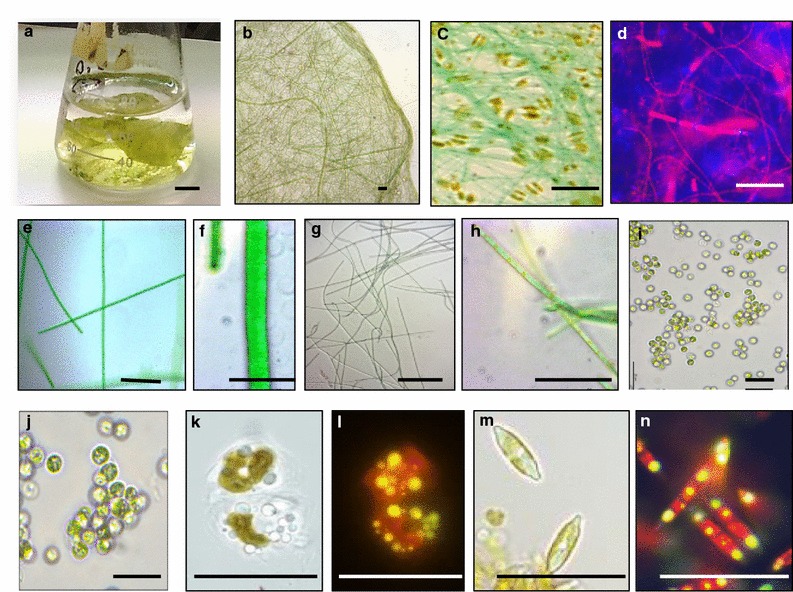
Biofilm #52 and its isolated photosynthetic components. **a** Biofilm #52 growing in F2 media in flask. **b**–**d** Biofilm #52 under different magnifications. **e**, **f** BAPS-52-1. **g**, **h** BAPS-52-2. **i**, **j** BAPS-52-3. **k**, **l** BAPS-52-5. **m**, **n** BAPS-52-4. **d** Under UV light, **l**, **n** staining for lipids with Nile Red. *Scale bars* **a** 1 cm, **b**–**n** 20 µm

**Fig. 2:**
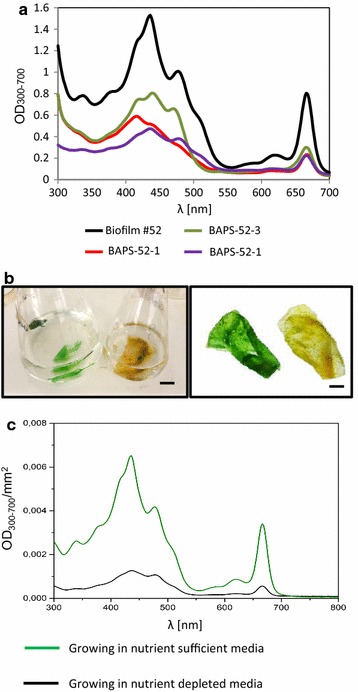
Absorbance spectra of Biofilm #52 and its photosynthetic components. **a** OD_300–700_ values of pigments extracted from Biofilm #52 and its photosynthetic components. **b** Biofilm #52 grown in nutrient-sufficient (*green*) and nutrient-depleted (*yellow*) media. **c** OD_300–700_ values of pigments extracted from Biofilm #52 grown in nutrient sufficient and nutrient-depleted media. **b** *Scale bar* 1 cm

Phenotypic and phylogenetic analysis of the Biofilm #52 isolated from a saline lake showed that it contains five biofilm-associated photosynthetic species (BAPS) which include two filamentous cyanobacteria, BAPS-52-1 and BAPS-52-2, one unicellular microalgae BAPS-52-3 and two diatoms, BAPS-52-4 and BAPS-52-5 (Fig. 1). Detailed characterization of these components is shown in Additional file 4: Figure S4A–E. Some of these representatives were also identified from the saline biofilms isolated from coastal lagoons in different parts of the world [46, 47]. Non-photosynthetic inhabitants were represented by two fungi, *Acremonium* sp. and *Aspergillus* sp. and a bacterium, *Bacillus stratosphericus*. The biomass growth of the photosynthetic biofilms was assessed by changes in absorption profiles (200–700 nm) of their extracted pigments. These profiles were compared with the fingerprint profiles of the isolated photosynthetic components [48]. Growing in F2 medium, the Biofilm #52 showed a complex absorption spectrum representing additive spectra of its photosynthetic inhabitants, with two main areas of absorption: 400–500 nm (blue light absorbing spectra) and around 680 nm (red light absorbing spectra) (Fig. 2a). Three major peaks around 430, 450, and 480 nm roughly correspond to chlorophyll A (ChlA, 430 nm), chlorophyll B (ChlB, 460 nm) and representatives of the carotenoids and phycobilins (450–550 nm), respectively. ChlA was found in the algal and cyanobacterial representatives [49, 50]. ChlB occurs only in “green algae” and phycobilins are found in cyanobacterial populations. A number of additional peaks between 300 and 500 nm likely correspond to the other photosynthetic antenna components, such as lutein, zeaxanthin, and lycopene, which are common also in brown algae and diatoms [51, 52]. Other characteristic absorption peaks of photosynthetic pigments were detected between 650 and 700 nm, which represent red light absorption spectra of ChlA (665 nm) and ChlB (652 nm). The level and composition of the pigments in the biofilm extracts varied significantly under different growth conditions and stresses, reflecting changes in the populations of the inhabitants. Growth in nutrient-depleted media led to rapid decolourization of biofilms as a result of a reduction in the concentrations of the pigments (Fig. 2b, c). We used decolourization of biofilms as a visual indicator of depletion of nutrients in growth media. In these studies, the value of the absorption peak of ChlA at 430 nm (OD_430_/mL) was used for quantification of the growth of photosynthetic biomass. This method allowed for assessment of biomass’ growth under small-scale conditions (growth in microtiter plates). When it was possible, the total biomass of biofilms and their components were also estimated by changes in dry weight.

**Fig. 3:**
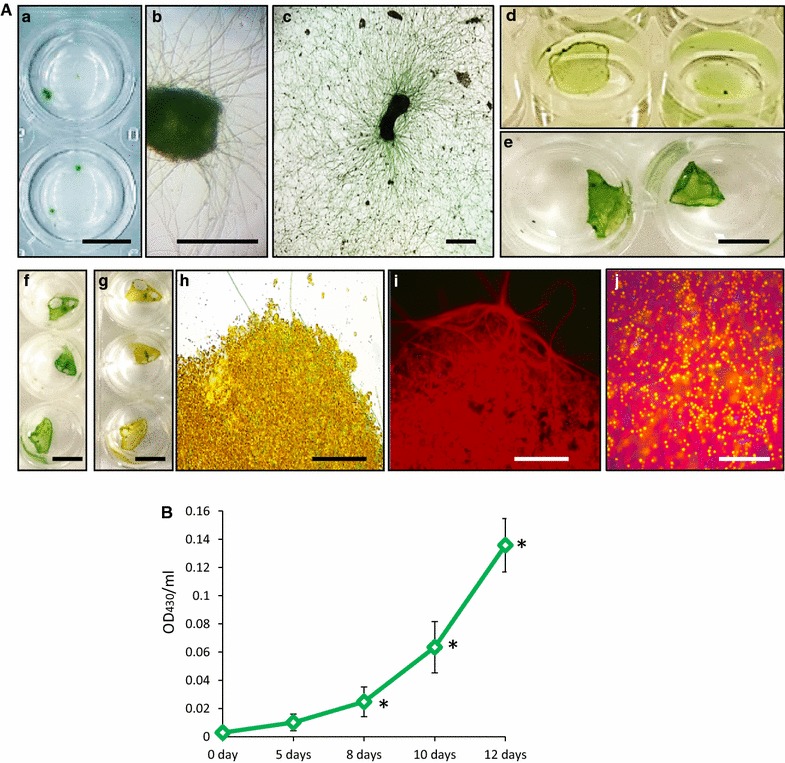
Growth of Biofilm #52 in microtiter plate. **A** 1 mm^2^ biofilm (seed culture) at day 0 (*a*, *b*); in 24 h (*c*); 7 days (*d*) and 9 days (*e*); days 9 and 14 (*f* and *g*, respectively); (*h*–*j*) growth of BAPS-52-4 and BAPS-52-5 diatoms within Biofilm #52 in F2 + Si media. (*i*) under UV light; (*j*) staining for lipids with Nile Red. *Scale bars* **A** (*a*, *d*, *e*–*g*), 1 cm; **A** (*b*, *c*), 1 mm; **A** (*h*–*j*), 20 µm. **B** Growth rates of Biofilm #52 in F2 media. Significance levels: *P < 0.05

For growth-rate assessments under small-scale conditions (2 mL microtiter plate’s wells), a 1-mm^2^ piece of Biofilm #52 (OD_430_/mL = 0.0029 ± 0.0015) was normally used as a ‘seed culture’ (Fig. 3A: a, b). The growth of filamentous cyanobacteria was observed within the first 24 h. Newly grown biofilms were detected floating on the surface of the F2 medium at days 7–8. Extended growth (over 14 days) in the same medium led to decolourization of the biofilm, obviously as a result of depletion of the main growth nutrients (Fig. 3A: f, g). The addition of silica to F2 medium boosted the growth of diatom components within Biofilm #52 (Fig. 3A: h–j). Quantification of the biofilm growth (OD_430_/mL) showed up to 46.6-fold increase of biomass after the first 12 days (Fig. 3B). This was correlated with production of 7.2 ± 1.5 mg dw/mL of biomass in each well, with biomass production rate of 0.6 ± 0.2 mg dw/mL-day (600 mg dw/L-day). Growing biomass for 1 month in 500 mL medium from the same seed culture led to a lower growth rate of 160 mg dw/L-day. This can be explained by gradual depletion of nutrients in the medium during the long-term experiment, which was observed by decolourization of the biofilm biomass (Additional file 5: Figure S5). Lower productivity (40–55 mg/L-day) was observed for growing monocultures of biofilm-forming cyanobacterial strains, such as *Trichormus variabilis, Anabaena augstmalis, Phormidium autumnale, Synechocystis aquatilis, Calothrix* sp., *Nostoc* sp. and *Trichormus variabilis* [53].

#### Attachments between photosynthetic inhabitants

Microscopic analysis of mixed representatives of photosynthetic inhabitants isolated from Biofilm #52 showed that they can strongly attach to each other growing in F2 media (Additional file 6: Figure S6). In general, the cell surfaces of microalgal and cyanobacterial representatives can be attached to each other via hydrogen bonds, hydrophobic (EPS) and electrostatic interactions [54, 55]. To understand the mechanisms of these interactions, we firstly evaluated the electrostatic charge distributions across the surfaces of isolated photosynthetic components and the Biofilm #52 grown in the F2 medium. Zeta potential values of all components in F2 (pH 6.9–7.2) showed strong negative surface charges between −10.5 (BAPS-52-5) and −23.1 mV (BAPS-52-2) (Additional file 7: Table S1). The ionic charge of intact Biofilm #52’s surface was also negative. These data suggested that production of biofilms cannot be explained solely by ionic interactions of their cellular components. A negative ionic charge of some of the cyanobacteria and microalgae has been shown previously [56, 57].

Detailed microscopic analyses of the photosynthetic components isolated from different biofilms showed the ability of some of them to secrete water-insoluble EPS (Additional file 6: Figure S6). This suggests that EPS matrix is mainly responsible for the attachment of all components of BAPS-52 to each other. As a result of these attachments, tailored novel biofilms can be assembled from the individual components isolated from different biofilms. This opens the possibility for a new strategy for bio-flocculation and concentration of commercial microalgal cells.

#### Filamentous cyanobacteria, BAPS-52-2-mediated flocculation, and concentration of ***Isochrysis*** cells

**Fig. 4:**
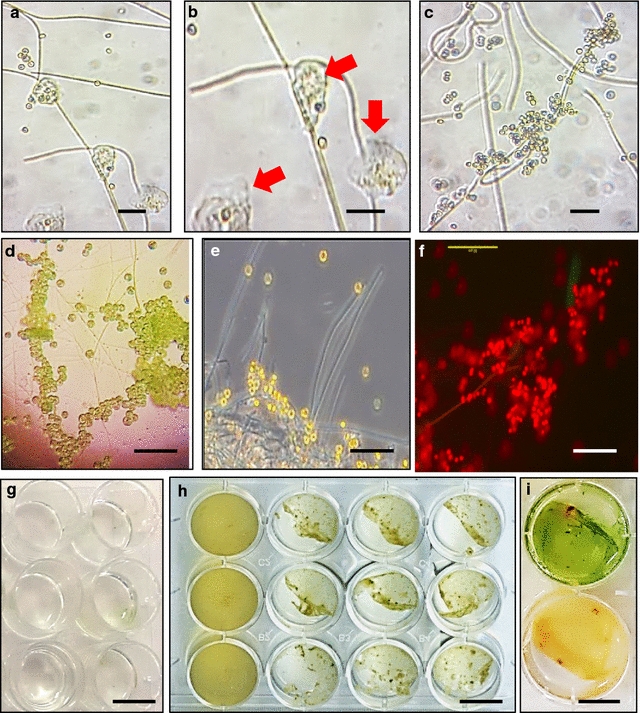
Bio-flocculation of *Isochrysis* sp. cells by BAPS-52-2 filaments. **a**–**f** Attachment of *Isochrysis* sp. cells to BAPS-52-2 filaments. Secreted EPS shown by *red arrows*. **g** *Isochrysis* cells (*left* wells, controls) and *Isochrysis* cells mixed with BAPS-52-2 filaments (*right* wells) at day 0 and day 10 (**h**). Green pigmentation produced by biofilm produced by monocultured BAPS-52-2 filaments at day 10 (**i** *upper* well) and Biofilm #102 at day 10 (**i** *bottom* well). *Scale bars* **a**–**f** 20 µm; **g**–**i** 1 cm

It was of interest to test whether the scaffold produced by BAPS-52-2 filaments and a matrix of their secreted EPS can be used for bio-flocculation and concentration of commercial microalgal species which are not natural inhabitants of biofilms. We co-cultured the different marine unicellular microalgae, *Isochrysis* sp., *Nannochloropsis oculata* (*N. oculata*) and *Nannochloris* sp., with filamentous cyanobacteria BAPS-52-1, BAPS-52-2 and a mixture of BAPS-52-1 and BAPS-52-2. This led to their attachment to filamentous cyanobacteria (Additional file 8: Figure S7). We have quantified the bio-flocculation capacity of filamentous cyanobacteria BAPS-52-2 by co-cultivating it with *Isochrysis* sp. cells. *Isochrysis* sp. are yellow–brown marine motile phytoflagellates. Being rich in oil, *Isochrysis* species are one of the few known haptophyte marine microalgae that can biosynthesize polyunsaturated long-chain (C37–39) alkenones, alkenoates, and alkenes (PULCA) [58]. Breaking their double bond long chains into 8–13 carbons using the technology of olefin metathesis is becoming increasingly popular as a new potential fuel source [59]. We did not find *Isochrysis* cells in 18 isolated saline biofilms. Zeta potential values for both of these components showed negative surface charges, −14.3 mV for *Isochrysis* and −23.2 mV for BAPS-52-2, indicating that this interaction cannot be explained by the simple electrostatic interaction between oppositely charged surfaces (Additional file 7: Table S1). Microscopic analysis of a co-culture of *Isochrysis* cells and cyanobacterial filaments showed their strong EPS-mediated attachment to each other (Fig. 4a–f).

**Fig. 5:**
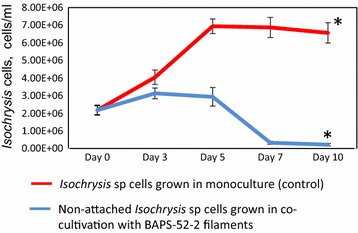
Bio-flocculation of *Isochrysis* sp. cells by BAPS-52-2 filaments. The *red line* shows *Isochrysis* growth in monoculture (control); the *blue line* shows a number of non-attached *Isochrysis* after co-cultivation with BAPS-52-2 filaments. Significance levels: *P < 0.05

We used two strategies for quantification of the flocculation efficiency of *Isochrysis* sp. cells. In the first one, *Isochrysis* cells (2.35 × 10^6^ cells/mL) were mixed with a seed culture of BAPS-52-2, OD_430_/mL = 0.02 ± 0.01 (day 0, Fig. 4g), and the mixture was grown for 10 days with slow shaking (50 rpm) microtiter plates. The number of *Isochrysis* cells in the medium non-attached to biofilm was counted and compared to the number of the monocultured *Isochrysis* cells grown under the same conditions (control). In the control experiment, the number of monocultured *Isochrysis* cells increased 3.5-fold over the first 5 days (Fig. 5). Co-cultivation of *Isochrysis* and BAPS-52-2 led to visible biofilm production at days 5–7, designated as Biofilm #102 (Fig. 4h). This was correlated with a reduction in the number of non-attached *Isochrysis* cells after day 5 to 2.1 × 10^5^ cells/mL, which is up to 11-folds lower than the starting *Isochrysis* cell density at day 0 (96% efficiency of flocculation) and 29-folds lower than the number of *Isochrysis* cells grown in the control experiment. Some reduction in the number of non-attached *Isochrysis* cells in the control experiment after day 5 can be explained by their attachment to each other growing in high-density suspension. With a final total biomass of the Biofilm #102 biofilm as 17.1 ± 5.1 mg/mL, the efficiency of flocculation/concentration of *Isochrysis* cells in this experiment was calculated as 2.53–3.73 × 10^5^ cells/mg dw of BAPS-52-2. Interestingly, the total biomass of Biofilm #102 was lower than the additive biomasses of both its components growing separately: BAPS-52-2, 25.5 ± 6.5 mg/mL and *Isochrysis* cells, 11.4 ± 3.7 mg/mL. This suggests that Biofilm #102 has consumed more nutrients than its components grown in monocultures. This is supported by the fact that after 10 days growth in monoculture, the BAPS-52-2 biofilm was still green, whereas Biofilm #102 had lost its pigmentation (Fig. 4i).

**Fig. 6:**
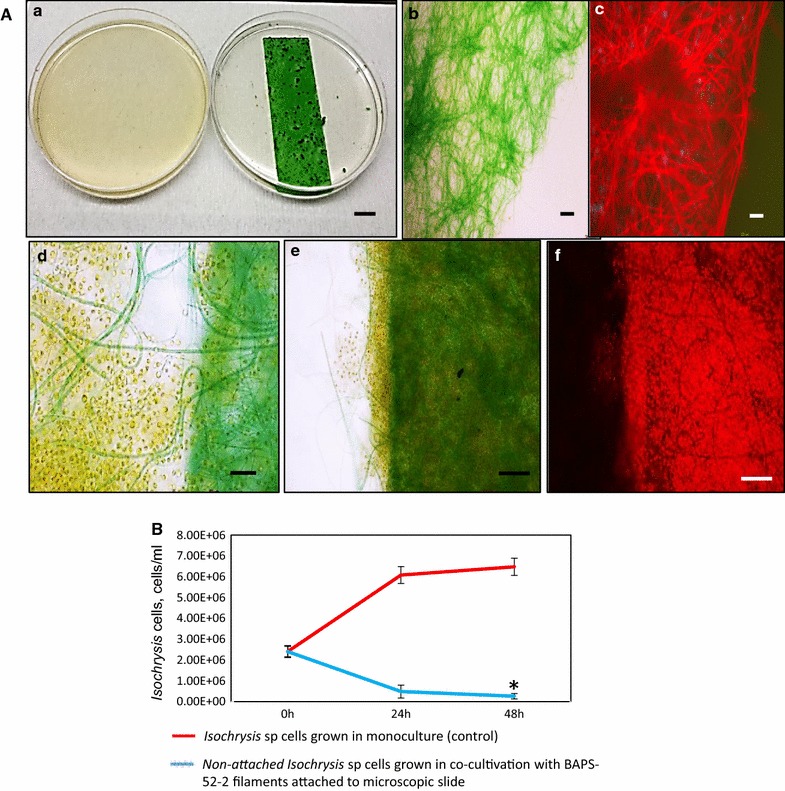
Bio-flocculation of *Isochrysis* sp. cells by BAPS-52-2 filaments. **A** (*a*) Culture of *Isochrysis* sp. cells at day 0 (*left*). The culture of *Isochrysis* sp. cells 2 days after co-culture with BAPS-52-2 filaments attached to the microscopic slide (*right*); (*b*, *c*) BAPS-52-2 filaments grown on the microscopic slide; the culture of *Isochrysis* sp. cells mixed with BAPS-52-2 filaments at day 0 (*d*) and day 2 (*e*, *f*). (*c*, *d*) images under UV light. *Scale bars* (*a*), 1 cm; (*b*–*f*), 20 µm. Significance levels: *P < 0.05. **B** *Red line* shows *Isochrysis* growth in monoculture (control); *blue line* shows a number of non-attached *Isochrysis* after co-cultivation with BAPS-52-2 filaments

In the second experiment, the BAPS-52-2-based biofilm was produced after growing in monoculture on the surface of a microscope slide (75 × 26 mm, 1.1 ± 0.4 g dw, Fig. 6A). This attached Biofilm #102 was placed in a Petri dish containing 20 mL of *Isochrysis* cells (2.4 × 10^6^ cells/mL) and co-cultured for 48 h. In the control experiment containing monocultured *Isochrysis* cells, the number of cells increased 2.7-fold over 48 h, reaching 6.5 × 10^6^ cells/mL (Fig. 6B). The number of *Isochrysis* cells after co-culture with the BAPS-52-2-based biofilm (Biofilm #102) were decreased up to 10-folds after 48 h of incubation, reaching 2.5 × 10^5^ cells/mL (90% compared to the number of *Isochrysis* cells at day 0). The efficiency of flocculation of *Isochrysis* cells was calculated as 4.3 ± 1.2 × 10^7^ cells/g dw of BAPS-52-2. However, since the *Isochrysis* cells were mainly attaching to the upper layer of the filamentous mat, the real flocculation efficiency should be higher. Both experiments showed that filamentous cyanobacteria secreting a matrix of EPS can be used for efficient flocculation of microalgal species which are not their natural co-inhabitants.

### Application of biofilms for wastewater treatment

#### Biofilm #52-mediated treatment of synthetic wastewater

For the bioremediation experiment, highly saline selenium-rich synthetic wastewater (SeSW) was prepared by mixing a high concentration of phosphates (PO_4_-P), 1.3 g/L and ammonia (NH_4_-N), 55 mg/L, and a moderate concentration of nitrates (NO_3_-N), 15 mg/L (Additional file 9: Table S2). This composition simulates the characteristics of saline effluents from typical textile dyeing, finishing and laundry-detergent producing industries [60, 61]. To increase the toxicity of this wastewater, selenium (Se) was added to a final concentration of 800 µg/L. This wastewater was toxic for the amphipods, such as *Allorchestes compressa* (*A. compressa*), normal inhabitant of the saltwater lakes killing 87% of them after the first 120 h of exposure (Additional file 10: Figure S8).

**Fig. 7:**
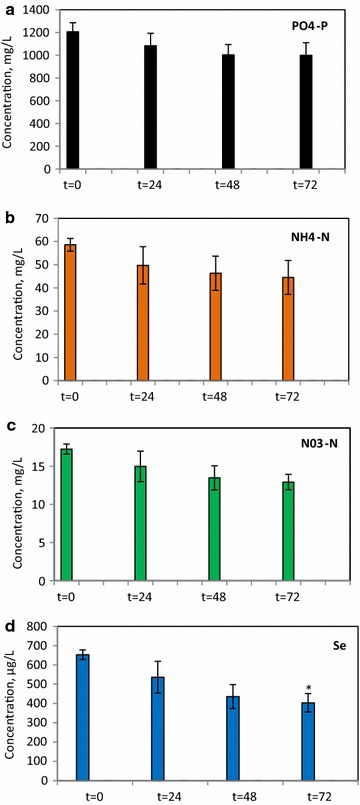
Reductions in concentrations of nutrients and selenium after treatments of SeSW with Biofilm #52. Reduction in concentrations of PO_4_-P (**a**); NH_4_-N (**b**); NO_3_-N (**c**) and Se (**d**). Significance levels: *P < 0.05

For the bioremediation experiment, three pieces of Biofilm #52 (round, 8.55 mm^2^, 1.8 ± 0.6 g wet weight/0.25 ± 0.1 g dw) were initially starved by growing for an extended time in growth medium until decolourization was observed (Additional file 11: Figure S9). In the bioremediation experiment, we used 3-day treatment of SeSW by starved Biofilm #52. Growth in SeSW led to green colourization of Biofilm #52 which was reflected by a significant increase in the concentration of all pigments absorbing in areas between 400 and 700 nm (not shown). The first 3 days of treatment of SeSW by Biofilm #52 did not lead to statistically significant changes (*P* ≤ 0.05) in the biofilm’s biomass (0.26 ± 0.15 g dw); however, it led to up to 24% uptake of NH_4_-N, 26% uptake of NO_3_-N and 17% uptake of PO_4_-P from SeSW (Table 1; Fig. 7). Uptake rates of the nutrients were 4.7, 69 and 1.4 mg/L-day for NH_4_-N, PO_4_-P, and NO_3_-N, respectively. There was 38% uptake of Se from the SeSW with absorption rates of 83.6 µg Se/L-day which correlates with the accumulation of 333.3 µg Se/g dw-day in Biofilm #52’ biomass (Table 1). Treatment of the SeSW with Biofilm #52 showed a reduction in the mortality rate of *A. compressa* from 87 to 47% indicating that the SeSw was significantly cleaner after 3 days of treatment (Additional file 10: Figure S8). In natural ecosystems, phototrophic biofilms representing a multilayered community of photoautotrophs and heterotrophs play a key role in the self-purification of aquatic ecosystems [62]. Application of microalgal biofilms as a post-treatment system for municipal wastewater showed nutrient loading rates from 1.5 to 1030 mg/L-day for TN and 1.7-160 mg/L-day for TP with biomass production rates 2.2–5.5 g/m^2^-day [32–37, 63, 64]. Most of these studies used a popular strategy which employs matrix-immobilized microalgae systems in which algal cells (usually represented by single microalgae) are grown on the surface of some solid-supporting materials. In these immobilized culture systems, the algal biomass can be naturally concentrated and easily harvested [21, 22].

**Table 1:** Reduction in concentrations of NH_4_, NO_3_, PO_4_ and Se in SeSW after treatment with Biofilm #52

Final biomass gDW/L	NH_4_ uptake				PO_4_ uptake				NO_3_ uptake				Se uptake			
	NH_4_, mg/L, final conc	NH_4_ uptake, %	NH_4_ uptake rate, mg/L-day	NH_4_ uptake, mg/gDW-day	${\text{PO}}_{{4^{- } }}\text{P}$, mg/L, final conc	PO_4_-P uptake, %	PO_4_-P uptake rate, mg/L-day	${\text{PO}}_{{4^{ - } }}\text{P}$ uptake, mg/gDW-day	${\text{NO}}_{{3^{ - } }}\text{N}$, mg/L, final conc	${\text{NO}}_{{3^{ - } }}\text{N}$ uptake, %	${\text{NO}}_{{3^{ - } }}\text{N}$ uptake rate, mg/L-day	${\text{NO}}_{{3^{ - } }}\text{N}$ uptake, mg/gDW-day	Se, µg/L, final conc	Se uptake, %	Se uptake rate, mg/L-day	Se uptake, mg/gDW-day
2.5 ± 0.1	44.5 ± 8.2	24.0 ± 8.9	4.7 ± 0.8	1.9 ± 0.1	999.56 ± 78.6	17.1 ± 4.9	68.8 ± 9.0	27.5 ± 5.3	11.9 ± 0.4	25.5 ± 3.8	1.4 ± 0.1	0.6 ± 0.1	403.7 ± 45.4	38.0 ± 8.2	83.6 ± 30.3	33.3 ± 7.8

#### Treatment of ROC with assembled Biofilm #109

**Fig. 8:**
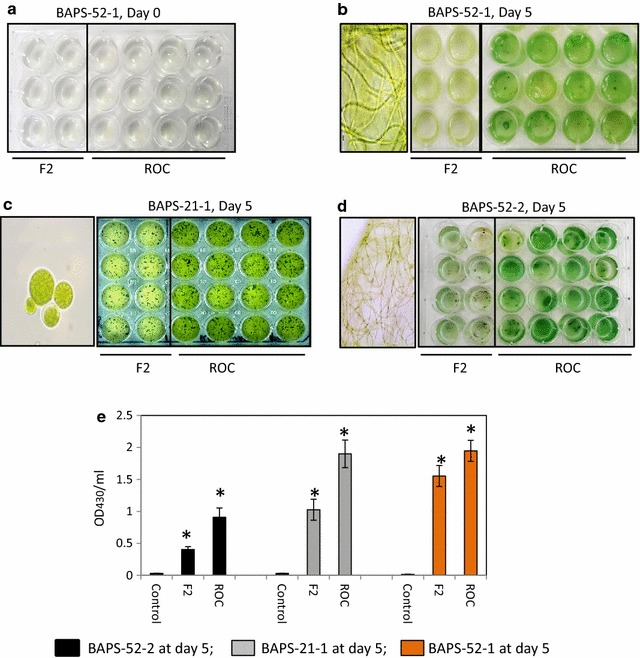
Growth of components isolated from Biofilms #52 and #21 in F2 media and ROC. **a** The growth of BAPS-52-1 in F2 media and ROC at day 0. Similar images were observed for concentrations of BAPS-52-2 and BAPS-21-1 at day 0 (not shown); Growth rates in F2 and ROC at day 5 for BAPS-52-1 (**b**), BAPS-21-1 (**c**), BAPS-52-2 (**d**). **e** Growths of BAPS-52-1, BAPS-21-1 and BAPS-52-2 after 5 days in F2 media and ROC. Control, concentrations of components in F2 and ROC at day 0. Significance levels: *P < 0.05

Saline ROC stream samples are high in concentrations of nutrients (mainly nitrates, phosphates), heavy metals and microelements, such as arsenic, manganese, and others (Additional file 12: Tables S3). We tested the effect of ROC on the growth rates of different saline biofilms and their isolated components by growing them for 5 days in microtiter plates containing ROC or F2 medium, as a control. Three representatives, BAPS-52-1 and BAPS-52-2 and unicellular green microalgae isolated from Biofilm 52 and BAPS-21-1 isolated from Biofilm #21 (Additional file 4: Figure S4) were selected because of their significant growth in ROC (Fig. 8a–d). Interestingly, they all showed higher growth rates in ROC than in F2 medium. The OD_430_/mL was valued 2.5-, 1.9- and 1.3-fold higher in ROC than in F2, for BAPS-52-1, BAPS-21-1, and BAPS-52-2, respectively. After 5 days growth in ROC, their biomasses were increased 45-, 95- and 135-fold for BAPS-52-1, BAPS-21-1, and BAPS-52-2, respectively (Fig. 8e).

**Fig. 9:**
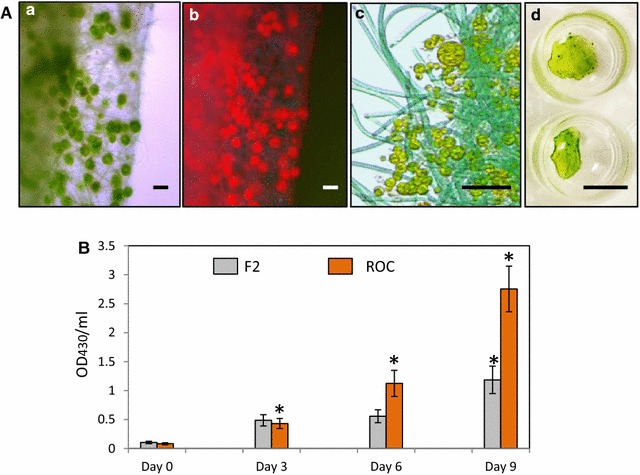
Growth of assembled Biofilm #109 in F2 media and ROC. **A** (*a*–*d*) Images of Biofilm #109 assembled from BAPS-52-1, BAPS-21-1 and BAPS-52-2; *Scale bars* **A** (*a*–*c*),20 µm; **A** (*d*), 1 cm. **B** Growth rates of Biofilm #109 after 9 days in F2 media and ROC. Significance levels: *P < 0.05

As the next step, three selected components, BAPS-52-1, BAPS-21-1, and BAPS-52-2, were mixed for the production of assembled biofilm (Biofilm #109) in ROC and F2 (Fig. 9A). As expected, Biofilm #109 grew faster in ROC than in F2 leading to a 35-fold increase in OD_430_/mL after day 9 (Fig. 9B). The intensive growth of Biofilm #109 in ROC was correlated with a significant reduction in concentrations of the key nutrients (Additional file 13: Table S4). The concentration of PO_4_ was reduced from 135 to 15 mg/L (89% uptake). NO_3_ was almost undetectable after 9 days of treatment (99% uptake). With a final biomass of the biofilms 0.0127 ± 0.007 mg dw/mL, this was correlated with absorption rates of PO_4_ and NO_3_ as 1.0 and 1.6 mg dw/L-day, respectively (Table 2; Additional file 13: Table S4). Concentrations of the heavy metals Mn, Fe, Ni, Cu and Pb were decreased by 3.6, 2.3, 1.3, 4.1 and 3.6-folds, respectively.

**Table 2:** Absorption rates of some of ROC components after treatment with Biofilm #109

Final biomass, mg DW	PO_4_ uptake				NO_3_ uptake				Fe uptake				Cu uptake			
	$\tt {\text{PO}}_{{4^{ - } }}P$, mg/L, final	PO_4_-P uptake, %	PO_4_-P uptake rate, mg/L-day	${\text{PO}}_{{4^{ - } }}\text{P}$ uptake, mg/gDW-day	${\text{PO}}_{{4^{ - } }}\text{P}$, mg/L, final	${\text{PO}}_{{4^{ - } }}\text{P}$ uptake, %	${\text{NO}}_{{3^{ - } }}\text{N}$ uptake rate, mg/L-day	${\text{NO}}_{{3^{ - } }}\text{N}$ uptake, mg/gDW-day	Fe, mg/L, final	Fe uptake, %	Fe uptake rate, mg/L-day	Fe uptake, mg/gDW-day	Cu, mg/L, final	Cu uptake, %	Cu uptake rate, mg/L-day	Cu uptake, mg/gDW-day
13.1 ± 1.2	15 ± 3.4	89 ± 21.0	13 ± 1.2	1 ± 0.2	0.5 ± 0.2	99 ± 21.2	21 ± 5.1	1.6 ± 0.2	0.85 ± 0.2	58.2 ± 10.9	0.13 ± 0.1	0.01 ± 0.01	0.05 ± 0.02	78.0 ± 10.2	0.02 ± 0.01	0.002 ± 0.001

### Biofilms as feedstocks for bioenergy production

#### Lipid production in Biofilm #52

Lipid production and fatty acid composition (measured as fatty acid methyl esters, FAMEs) of Biofilm #52 and its four photosynthetic components are shown in Table 3. The lipid yield of Biofilm #52 grown in nutrient-sufficient F2 medium was estimated as 6.3 ± 1.2% of its dw biomass. In general, the fatty acid composition of Biofilm #52 reflects the averaged composition of its photosynthetic inhabitants, which is dominated by saturated fatty acids (SAFAs, 41%), represented mainly by lauric (C12:0), palmitic (C16:0) and stearic acid (C18:0) acids. Palmitic acid was the most abundant fatty acid found in all photosynthetic components of Biofilm #52. Interestingly, much higher, 33–44% of lauric acid (C12:0) was observed earlier in three representatives of *Oscillatoria* sp. [65]. The content of up to 31% of polyunsaturated fatty acids (PUFA) in Biofilm #52 was mainly contributed by linoleic (C18:2) and linolenic (C18:3) acids, which were high in BAPS-52-1 and BAPS-52-2. 4.2% of omega 3-containing, eicosapentaenoic acid (EPA) and 1.1% of docosahexaenoic acid (DHA), detected in Biofilm #52 were likely contributed by diatoms since these PUFA were absent for all other components. Despite the fact that both diatoms are very high in palmitoleic acid (C16:1), this was not strongly reflected in the composition of Biofilm #52 grown in nutrient-sufficient F2 medium. The monosaturated fatty acids (MUFA) represented just 16% of total FAMEs. Similar fatty acid compositions were described earlier for representatives of *Fistulifera* sp. and *Nitzschia* sp. [66, 67].

**Table 3:** Lipid level and composition in biofilms and their isolated components

Lipids/fatty acids	Formulas	Biofilm #52	BAPS-52-1	BAPS-52-2	BAPS-52-3	BAPS-52-4	BAPS-52-5
Lipid concentration		6.3 ± 1.2	5.2 ± 1.0	6.3 ± 1.8	6.9 ± 2.1	25.1 ± 4.8	20.2 ± 5.2
Lauric acid	C12:0	4.3 ± 0.9	10.0 ± 2.2	3.0 ± 0.8	2.0 ± 0.7	ND	ND
Myristic acid	C14:0	1.3 ± 0.2	ND	1.2 ± 0.3	2.2 ± 0.4	6.1 ± 1.9	5.2 ± 1.3
Palmitic acid	C16:0	28.3 ± 5.1	27.1 ± 6.2	26.8 ± 4.8	28.1 ± 6.0	28.6 ± 6.0	31.0 ± 6.0
Stearic acid	C18:0	7.01 ± 1.8	4.0 ± 0.9	3.3 ± 0.7	21.8 ± 5.0	3.2 ± 0.8	1.2 ± 0.3
SAFAs		40.9 ± 8.5	41.1 ± 8.2	34.3 ± 6.8	53 ± 10.6	37.9 ± 7.2	37.4 ± 6.8
Palmitoleic acid	C16:1	7.4 ± 1.6	0.2 ± 0.1	8.9 ± 2.1	8.8 ± 1.6	31.1 ± 7.2	33.0 ± 6.3
Oleic acid	C18:1	8.5 ± 1.9	5.2 ± 1.3	16.8 ± 4.0	5.4 ± 1.3	1.6 ± 0.3	2.1 ± 0.5
Gondoic acid	C20:1	ND	ND	0.5 ± 0.2	ND	ND	ND
MUFAs		15.9 ± 4.0	5.5 ± 1.3	26.2 ± 5.3	13 ± 3.1	32.7 ± 7.1	35.1 ± 6.0
Hexadecadienoic acid	C16:2	3.7 ± 0.5	4.5 ± 1.0	0.6 ± 0.1	ND	3.1 ± 0.9	1.1 ± 0.2
Linoleic acid	C18:2	12.1 ± 2.8	16.2 ± 4.2	11.4 ± 3.9	8.0 ± 2.8	0.8 ± 0.2	0.9 ± 0.2
Linolenic acid	C18:3	9.8 ± 2.1	15 ± 3.9	11.5 ± 3.2	12.6 ± 3.0	0.7 ± 0.3	ND
Arachidonic acid	C20:4	ND	ND	ND	ND	5.0 ± 2.8	2.5 ± 1.8
Eicosapentaenoic acid (EPA)	C20:5	4.2 ± 1.0	ND	ND	ND	12.1 ± 4.4	15.0 ± 4.9
Docosahexaenoic acid (DHA)	C22:6	1.1 ± 0.3	ND	ND	ND	4.1 ± 1.9	3.1 ± 1.5
PUFAs		30.9 ± 6.9	35.7 ± 7.5	23.5 ± 5.0	20.1 ± 4.2	25.8 ± 5.1	22.6 ± 4.0
Others		12.3 ± 4.2	17.7 ± 5.8	15.8 ± 3.8	14 ± 4.1	3.3 ± 1.1	4.9 ± 1.9

**Fig. 10:**
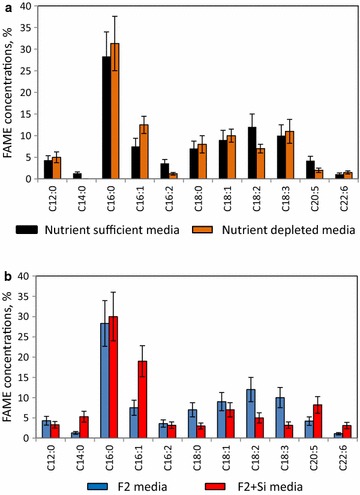
FAME concentrations of Biofilm #52. **a** FAME concentrations of Biofilm #52 grown in nutrient-sufficient and -depleted F2 media. **b** FAME concentrations of Biofilm #52 grown in F2 and F2 + Si media

Starving in the nutrient-depleted medium when the green pigmentation had converted into the light-brown led to some increase in lipid content up to 8.1 ± 2.4% of dw. The nutritional deficiency resulted also in some changes in the FAME composition (Fig. 10a). In particular, the proportion of total polyunsaturated FAME decreased from 31 ± 5.2% to 22.7 ± 3.2% mainly because of reductions in the proportions of hexadecadienoic acid (C16:2), linoleic acid (C18:2) and EPA (C20:5). The proportion of total monounsaturated FAME increased from 16 ± 3.8% to 22.4 ± 5.7%, due to an increase in C16:1. The ability of individual microalgal representatives to increase their lipid level and modify fatty acids’ compositions under nutritional stresses were previously shown (for reviews see [2–4]). The addition of silica to F2 medium boosted the growth of diatoms increasing the total lipid yield to 15.3 ± 4.0% dw (Figs. 3A: h–j, 10b). As expected, the proportion of diatom-specific FAMEs, C16:1, C20:5 and C22:6, was increased compared with Biofilm #52 growing in F2. The highest increase in concentrations was observed for palmitoleic acid (C16:1) and EPA (C20:5). The proportions of cyanobacteria-specific FAMEs, C12:0, C18:2 and C18:3, however, were reduced.

Bio-flocculation of *Isochrysis* cells by BAPS-52-2 (Biofilm #102) led to the production of biomass with the lipid content of 14% dw, which is lower than that in monocultured *Isochrysis* cells but much higher than the lipid content of the natural Biofilm #52 (Table 4). The proportion of PUFA in Biofilm #102 was increased from 23 to 35% compared with BAPS-52-2 as a result of the presence of polyunsaturated omega-3 fatty acids, arachidonic acid (C20:4), eicosapentaenoic acid (C20:5) and docosahexaenoic acid (DHA, C22:6).

**Table 4:** Lipid level and composition in assembled Biofilm #102 and its components

Lipids/fatty acids	Formulas	BAPS-52-2	*Isochrysis*	Biofilm #102
Lipid concentration		6.3 ± 1.8	18.3 ± 4.8	14.3 ± 4.2
Lauric acid	C12:0	3.0 ± 0.8	5.0 ± 2.0	4.8 ± 2.1
Myristic acid	C14:0	ND	11.2 ± 2.8	8.8 ± 2.8
Palmitic acid	C16:0	26.8 ± 4.8	15.4 ± 3.1	21.9 ± 5.1
Stearic acid	C18:0	3.3 ± 0.7	ND	ND
SAFAs		33.1 ± 6.8	31.6 ± 8.2	35.5 ± 8.9
Palmitoleic acid	C16:1	8.9 ± 2.1	5.1 ± 2.0	6.3 ± 2.8
Oleic acid	C18:1	16.8 ± 4.0	15.7 ± 6.2	13.99 ± 4.0
Gondoic acid	C20:1	0.5 ± 0.2	4.0 ± 2.0	3.25 ± 0.9
MUFAs		26.2 ± 5.3	24.8 ± 8.8	23.5 ± 9.3
Hexadecadienoic acid	C16:2	0.6 ± 1.0	3.8 ± 1.2	2.9 ± 1.0
Linoleic acid	C18:2	11.4 ± 3.9	ND	9.4 ± 0.8
Linolenic acid	C18:3	11.5 ± 3.2	7.0 ± 2.8	9.2 ± 2.9
Arachidonic acid	C20:4	ND	4.3 ± 2.0	2.3 ± 0.8
Eicosapentaenoic acid (EPA)	C20:5	ND	11.1 ± 2.9	8.2 ± 1.9
Docosahexaenoic acid (DHA)	C22:6	ND	4.2 ± 1.2	3.2 ± 1.0
PUFAs		23.5 ± 5.0	30.4 ± 6.0	35.2 ± 8.2
Others		17.2 ± 5.8	13.2 ± 4.2	5.8 ± 2.0

**Fig. 11:**
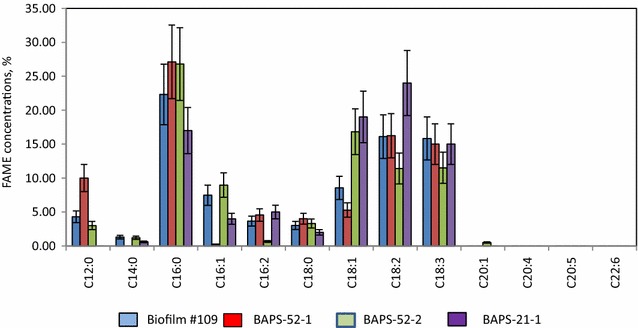
FAME concentrations of assembled Biofilm #109 and its components

Analysis of the lipid level and composition of the Biofilm #109 collected after treatment of ROC streams showed a total lipid yield of 5.8% dw fatty acids with the composition which was reflected the average composition of its three photosynthetic inhabitants (Fig. 11).

The chemical composition of biodiesel is dependent on the length and degree of unsaturation of the FAME chains. In general, the degree of unsaturation correlates with some key biodiesel parameters, such as an iodine number, cetane number, density and pour point [68]. Saturated feedstocks (such as those derived from coconut, palm and tallow) excel in cetane number and oxidation stability, but exhibiting poor cold flow properties, higher kinematic viscosity, flash point and lower heating value. The increase in the unsaturation degree decreases some these values, but improves the cold flow properties and increases moderately the heating value.

FAME composition of the natural and assembled biofilms were analysed for biodiesel properties using BiodieselAnalyzer© Ver. 2.2 (available on “http://www.brteam.ir/biodieselanalyzer“) and data published by [68] which is based on 26 different biodiesel feedstocks, comprising of 22 edible and non-edible vegetable oils and four animal fats. Table 5 shows the most important physical and chemical properties of biodiesels such as iodine value (IV), cold flow plugging point (CFPP), cetane number (CN), kinematic viscosity at 40 °C (ν), and others comparing them with data obtained for the main feedstocks used in the USA and the European countries for biodiesel production, canola, jatropha, rapeseed, palm and soybean [68].

**Table 5:** Biodiesel properties of biofilms

	Biofilm #52	Biofilm #52 + Si^a^	Biofilm #109	Biofilm #102	Feedstock^b^		Comments
					Min	Max	
SFA	40.9	41.9	31.6	38	8	45	
MUFA	15.9	26	16	23	41	60	
PUFA	23	16	32	24.1	10	36	
SV	169.7	170	168.6	146			
IV	68.7	76.4	87.7	86.9	57	111.7	124, upper limit in the EU^c^
CN	62.9	65.6	58.9	58.3	54	61.2	47, lower limit in the USA^c^
LCSF	6.3	4.5	4.2	1.9			
CFPP	3.4	−2.3	−3.2	−10.5	−11	11.4	
CP	9.8	10.7	6.5	5	−3.3	13	
PP	3.9	4.9	0.3	0.3	−9.8	11.8	
APE	57.5	38	72.6	74.1			
BAPE	37	26	47.8	48.5			
OS	7.95	17.3	6.3	8.9			
*υ*	2.7	2.8	2.6	2.9	4.4	4.6	1.8, lower limit in the USA^a^
*ρ*	0.69	0.74	0.7	0.75	0.87	0.89	0.86, lower limit in the EU^a^

*SFA* saturated fatty acid (%), *MUFA* mono unsaturated fatty acid (%), *PUFA* poly unsaturated fatty acid (%), *SV* saponification value (mg/g), *IV* iodine value, *CN* cetane number, *LCSF* long-chain saturated factor, *CFPP* cold filter plugging point (°C), *CP* cloud point (°C), *PP* pour point (°C), *APE* allylic position equivalent, *BAPE* bis-allylic position equivalent, *OS* oxidation stability (h), *υ* kinematic viscosity (mm^2^/s), *ρ* density (g/cm^3^)

^a^Growing Biofilm #52 in F2 + Si

^b^Feedstock: canola, jatropha, rapeseed, palm and soybean

^c^Giakoumis et al. [68]

One of the most influential properties of the diesel fuel is the dimensionless cetane number (CN), which represents the ignitability of the fuel, particularly critical during cold starting conditions. Low cetane numbers lead to long ignition delay, i.e. the long time between fuel injection and the start of combustion [69, 70]. European and US specifications dictate a cetane number of (bio)diesel fuel of at least 51 and 47, respectively. All biofilms showed CN values over 51.

The iodine number (IN, or iodine value IV), is a parameter used to determine the degree of unsaturation in a vegetable oil or animal fat [71]. The (average) iodine values of the examined feedstocks range from 7.8 (for the most saturated ME, coconut) to 184.5 (for the most unsaturated one, linseed), with an overall average value of 98.4. Canola, jatropha, rapeseed, palm and soybean showed IV value between 57 and 111.7, which is similar to the IV values observed for all analysed biofilms. Predicted biodiesel fuel properties of biofilms were also similar to the other physicochemical values DU, SV, LCDF, CFPP, CP, APE, BAPE, HHV and μ approved in many countries [68] (Table 5). The density of liquid was only biodiesel property which was below limits established for the US and the EU countries. Biofilm #102 saturated with *Isochrysis* cells showed the highest density value (0.74 g/cm^3^). Interestingly, enrichment of growth media with silica which promoted the growth of diatom components in Biofilm #52 led to changes the degree of unsaturation of fatty acids, which improved IV value from 68.7 to 76.4 and, importantly, also increased density value from 0.69 to 0.74 g/cm^3^.

#### Bio-hydrogen production from Biofilm #52

##### Batch fermentative biohydrogen production from acid-treated prehydrolysate of Biofilm #52

Biofilm #52 was used as a feedstock for bio-hydrogen production. Two treatment methods were used: acid pretreatment with 1% sulphuric acid and set up was kept in an autoclave for 60 min at 121 °C and enzymatic saccharification of a pretreated solid biomass (released after acid treatment) fraction with Cellic CTec2 (cellulase complex, Novozyme). The acid-pretreated solid fraction of biofilm biomass (5%) was enzymatically treated with the above-mentioned enzyme under conditions of 50 °C and agitation (180 rpm) for 24 h. In the former strategy, fermentative hydrogen production with *Enterobacter cloacae* (*E. cloacae)* was conducted in reactors containing anaerobically prepared BSH medium [44] supplemented with different amounts of acid-treated prehydrolysate (10–40% v/v).

Maximum hydrogen production (30.26 mmol/L) was observed from 10% (v/v) prehydrolysate containing 4.3 g/L of reduced sugars. With the increasing concentration of prehydrolysate, hydrogen production decreased, and no hydrogen production was observed from the fermentation broth supplemented with 40% biofilm prehydrolysate, containing 17.2 g/L of reduced sugars. *E. cloacae* DT-1 cells could not grow well at this concentration, and hydrogen production was not detected from 40% v/v prehydrolysate concentration. This could be attributed to higher concentration of acetic acid and other potential inhibitors generated during acid-pretreatment process. Acetic acid was the major metabolite generated during hydrogen production. Along with this, ethanol production was observed but in very less concentration. Hydrogen-yield efficiency achieved from acid-treated Biofilm #52 prehydrolysate was 1.81 mol of H_2_/mol of total reduced sugar (Table 6).

**Table 6:** Hydrogen yield and production from acid-treated Biofilm #52 prehydrolysate

Acid-treated biofilm prehydrolysate concentration (%)	Hydrogen production, (mmol/L)	Total sugar concentrations (g/L)	Acetic acid concentration (g/L)	Hydrogen yield, (mol of H_2_/mol of reduced sugar)
10	30.26	4.3	0.035	1.81
20	0.42	8.6	0.0539	0.075
30	0.39	12.9	0.085	0.070
40	Nil	17.2	0.076	Nil

##### Batch fermentative biohydrogen production from acid-treated Biofilm #52 prehydrolysate under decreased partial pressure of hydrogen

**Fig. 12:**
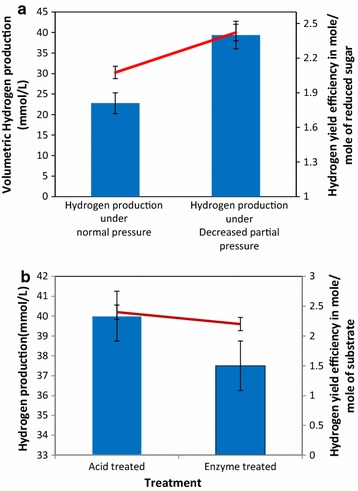
Hydrogen production from Biofilm #52 biomass. **a** Batch fermentative hydrogen production performance of *E. cloacae* DT-1 from acid-treated prehydrolysate and enzymatically saccharified Biofilm #52 sugar, under normal and reduced partial pressure. **b** Comparative hydrogen production performance of *E. cloacae* DT-1 from acid-treated and enzymatically hydrolysed biofilm biomass sugar, during the dark fermentation process

Hydrogen partial pressure has an inhibitory effect on hydrogen production during fermentation. Hence, to evaluate the hydrogen production performance of *E. cloacae* DT-1 strain from acid-treated Biofilm #52’s biomass, experiments were further conducted under decreased partial pressure of hydrogen achieved by removing the biogas by water displacement method from the bioreactor as soon as it is generated during fermentation [42].The performance of the 2 L scale batch [44] fermenter system was evaluated regarding volumetric hydrogen production in mmol/L and mL/L and hydrogen yield efficiency was presented in mol of H_2_/mol of reduced sugars. Fermentation of 10% of acid-treated Biofilm #52 by *E. cloacae* DT-1 under decreased hydrogen partial pressure led to 1.32-fold increase in hydrogen production, from 30.26 to 40 mmol/L (Fig. 12a). Hydrogen yield efficiency was increased from 1.8 mol of H_2_/mol of reduced sugars to 2.4 mol of H_2_/mol of reduced sugars. During fermentative hydrogen production, final pH decreased from 7.5 to 5.4. This is mainly because of accumulation of acetic acid (45 g/L), which was the major metabolite produced during hydrogen production from this biofilm prehydrolysate reduced sugar. Butyric acid production was not observed. These results imply that *E. cloacae* DT-1 strain most probably followed acetate pathway for hydrogen production from biofilm prehydrolysate reduced sugars.

##### Fermentative hydrogen production from enzymatically saccharified biomass sugars of Biofilm #52

In the second process, the acid-pretreated solid fraction of Biofilm #52 biomass was enzymatically saccharified by the cellulase complex (Cellic CTec2, Novozyme) before subjecting it to the *E. cloacae*-mediated fermentation. Saccharification of 10% of acid-treated Biofilm #52 led to the production of 6 g/L of reduced sugars, which after fermentation by DT-1 strain produced around 37.5 mmol/L of hydrogen with a hydrogen yield efficiency of 2.22 mol of H_2_/mol of reducing sugars. The final pH of the fermentation broth dropped down from 7.5 to 5.1 during hydrogen production. In this case also, acetic acid was the major soluble metabolite produced during fermentative hydrogen production. Along with this, butyric acid and ethanol production was also observed during hydrogen production in lower concentration.

Figure 12b demonstrates the hydrogen production and yield obtained from both acid-pretreated hydrolysate and enzymatically saccharified Biofilm #52 biomass. Overall, hydrogen yield efficiency of *E. cloacae*, DT-1 strain from biofilm #52 biomass, was 2.3 ± 2 mol of H_2_/mol of the substrate.

This data implies that biomass of the natural Biofilm #52 representing complex consortia of unicellular and multicellular (filamentous) photosynthetic and heterotrophic components, (cyanobacteria, green microalgae, diatoms, bacteria, and fungi), could be effectively used as a feedstock for production of biohydrogen by *E. cloacae* DT-1. The acid-treated biofilm biomass that contained 3.4 g/L of xylose-rich reduced sugar was also significantly utilized by *E. cloacae* DT-1 as this strain has xylose utilization property. *E. cloacae* DT-1 utilized the carbohydrates from complex biofilm biomass and produced hydrogen close to the value obtained by thermophilic anaerobic microbial consortium from pretreated algae biomass [72]. DT-1 strain, as mesophilic facultative anaerobe demonstrated higher hydrogen yield efficiency in mesophilic condition from xylose. The advantage of this strain is that it can not only utilize xylose but also can utilize glucose from the biofilm biomass even in the presence of protein and other EPS in mesophilic condition, Around 60% of maximum theoretical hydrogen yield efficiency (4 mol/mol of hexose sugar) was obtained by *E. cloacae* DT-1 from treated biofilm biomass, in mesospheric fermentation condition. Till date, this is the first report on biohydrogen production from treated biofilm biomass by a pure mesospheric *Enterobacter* sp.

## Conclusions

Algal/cyanobacterial-based biofilms are getting increasingly popular as an alternative to suspension-based culture systems because of their low-cost algal harvesting [21, 73–75]. Moreover, in algal biofilm systems, high algal concentration can be achieved with a significantly reduced amount of medium compared to the same biomass grown in suspension cultures [21, 22]. In this study, multidisciplinary research was applied to analyse different aspects of natural algal/cyanobacterial-based biofilms, their compositions, growth rates in response to the changing environmental conditions, as well as the interaction between individual inhabitants and assessments of their cumulative contributions to biofilm’s stability and chemical composition. We showed that the level and composition of photosynthetic inhabitants within biofilms could be tailored for the production of novel biofilms which are stable when growing in the new environmental ecosystems. These assembled biofilms can be specifically used for the (i) concentration of commercial microalgal species, working as bio-flocculating agents; (ii) for high-efficiency and low-energy strategies of treatment specific types of commercial wastewaters, and (iii) as novel sustainable feedstocks with compositions suitable for production of renewable bioenergy: bio-hydrogen and biodiesel.

## Methods

### Culture conditions

We have isolated and characterized a number of natural microalgal biofilms from the saline lakes and marine habitats around Melbourne, Victoria, Australia. Most of the samples were collected in mouth of rivers, where rivers meet an ocean: a mouth of Anglesea river, Victoria, Australia at the mouth of the Great Australian Bight (GPS coordinates of the location: 38°24′02.3″S 144°11′02.1″E) and Barwon river GPS coordinates of the location: 38.275747, 144.496864). Biofilms collected from saline lakes were washed with sterile F2 medium and cultured in 200-mL Erlenmeyer flasks at a photosynthetic photon flux density of 18 μmol photons m^−2^/s^−1^ with light:dark regime of 12:12 h and 20–22 °C temperature. Individual photosynthetic inhabitants were separated under the microscope and grown on F2 agar plates containing a set of antibiotics. Individual components were sub-cultured to other plates, and final axenic, bacterial and fungal-free strains were grown in liquid F2 medium without antibiotics. For purification of cyanobacterial strains, biofilms were grown on plates containing imipenem (100 µg/mL), kanamycin (5 μg/ mL) and nystatin (100 µg/mL). For green algae and diatoms, axenic cultures were established using a cocktail of antibiotics containing kanamycin (100 µg/mL), ampicillin (40 µg/mL), penicillin G (50 µg/mL), streptomycin (50 µg/mL) and nystatin, (100 µg/mL). Colonies were picked with a bacterial loop and re-streaked onto fresh plates containing cocktails of antibiotics. Light microscopy was used to characterize cell sizes and shapes under bright-field conditions using a Leica DM 2500 with the attached camera, a Leica DFC 310 FX. For biomass analysis, cultures were centrifuged at 6000*g* and then washed twice with sterile water, centrifuged again and dried at 65 °C.

### Pigment extraction and spectroscopy

Growing liquid cultures were centrifuged for 5 min at 12,000 g in a microcentrifuge and resuspended in 500 μL of ethanol. After centrifugation at 12,000*g* for 5 min, absorbance spectra of the supernatant were recorded using a POLARstar Omega Plate Reader Spectrophotometer (BMG LABTECH): from 200 to 800 nm.

### Genotyping

All isolated biofilm-associated components were identified phylogenetically to generate maximum-parsimony and distance trees using partial 16S, 18S, 23S and 28S rRNAs amplified with a set of primers for cyanobacteria, green algae, diatoms, fungi/yeast and bacteria described in Iteman et al. [76] and algae and cyanobacteria described by Di Pippo et al. [77]. The sequences of the primers are given in Additional file 14: Table S5. Amplified products were purified using the QIAquick PCR Purification Kit (Qiagen, Hilden, Germany). PCR products were run on agarose gel and appropriate bands were excised, and DNA extracted using the QIAquick Gel Extraction Kit (Qiagen, Hilden, Germany). DNA samples were directly sequenced in both directions by a commercial sequencing laboratory (AGRF, http://www.agrf.org.au/). The rRNA gene sequences were analysed using the BLAST function of GenBank at the National Center NCBI electronic site (http://www.ncbi.nlm.nih.gov/). Sequences were compared to those of the other strains obtained previously [78] and those from GenBank. Multiple sequence alignments were conducted with the CLUSTAL W at http://www.ebi.ac.uk/clustalw/index.html. MEGA7 (Molecular Evolutionary Genetics Analysis version 7.0) was used to generate maximum-parsimony and distance trees [79]. The gene sequences obtained in this study were deposited in the NCBI gene bank with following accession numbers: KX863346, KX863347, KX863348, KX863349, KX863350 and KX863351 for BAPS-52-1, BAPS-52-2, BAPS-52-3, BAPS-52-4, BAPS-52-5, and BAPS-21-1, respectively.

### Nile Red staining

For Nile Red staining, the cells were collected by centrifugation and re-suspended in 1 mL of 20% DMSO containing 5 μL of Nile Red stock solution (0.10 mg/mL of Nile Red dissolved in acetone) and incubated at 37 °C. The stained pellets were then subjected to fluorescence microscopy analysis to observe the formation of lipid droplets in the co-cultivated cells using Leica DM 2500 with an attached camera: Leica DFC 310 FX. Nile-Red filter: excitation at 543 nm, emission 555–650 nm.

### Lipid yield and fatty acid profile analysis

Extraction and analysis of lipid yield and FAME composition analysis of algal and cyanobacterial strains were performed using a method previously described the Folch method [80]. The FAME samples were analysed by GC [56].

### Zeta potential and cell size measurements

The zeta potential and cell size measurements of cells were obtained using a Nano-ZS/ZEN 3600. The zeta potentials were evaluated at a room temperature of 20 ± 2 °C. For each species, triplicate cultures were taken for measurements, and each dataset, 10–20 readings were taken for each sample [20].

### Synthetic wastewater treatment

The composition of saline SeSW is shown in Additional file 9: Table S1. For bioremediation experiments, three pieces of Biofilm #52 (round shape, 8.55 mm^2^, 1.8 ± 0.6 g wet weight/0.25 ± 0.1 g dw) were preliminarily starved by growing for an extended period in growth medium until decolourization (Additional file 11, Figure S9). These biofilms were used for the treatment of 100 mL of SeSW for 3 days. The containers were placed in 23 °C growth chamber with a 16-h photoperiod and a photosynthetic photon flux density of 50 µmol/m^2^/s. The solution in each container was mixed every day. Solution samples were analysed for ammonia cations, nitrate and phosphate anions by Dionex ICS-1100 (Thermo Scientific, USA).

### Selenium and heavy metal analysis measurements

SeSW samples were collected at the beginning and the end of the experimental period, filtered at 0.45 µm, acidified with concentrated HNO_3_ to pH 2 and kept at 4 °C.

Samples were diluted to 10 mL with Milli-Q water and analysed for total selenium concentration by inductively coupled plasma mass spectrometry (ICP-MS). The ICP-MS analysis was performed using an Agilent 7700x quadrupole-type ICP-MS (Agilent Technologies, Mulgrave, VIC, Australia) equipped with an Agilent ASX-520 auto sampler. The instrument was operated in He-mode. The integration time was 0.3 s per mass, 1 point per mass, 3 replicates and 100 sweeps per replicate.

### Amphipod acute toxicity test

Adult marine amphipods, *Allorchestes compressa*, collected from beaches at Queenscliff (Victoria, Australia) were used for the ecotoxicological test. These animals were maintained in 20-L glass tanks containing filtered seawater (salinity, 27.0–33.8%) at 24 °C, under a 16:8-h light:dark photoperiod with a light intensity of 400– 600 lx. The shrimps were fed with seagrass. However, they had not been fed for 24 h before the commencement of the tests. The acute toxicity tests were performed under static conditions in 500-mL glass beakers. Mortality (%) was assessed every 24 h up to 120 h and used to compare the toxicities between untreated and treated SeSW over time. The amphipods were considered as dead if they are not moving for 1 min.

### Bio-hydrogen production

#### Microorganism, media and growth condition

*Enterobacter cloacae* DT-1 (Gene Bank accession number: JX885522) isolated previously from crude oil-contaminated soil samples were collected from an oil refinery. This strain was routinely maintained anaerobically in BSH medium [44]. The pH of the BSH medium was adjusted to 7.5, and the incubation temperature was set at 37 °C.

#### Acid treatment of biofilm biomass

Ten grams of dried *biofilm* biomass sample was hydrolysed in 1% sulphuric acid by autoclaving for 60 min at 120 °C. The hydrolysed biomass was centrifuged at 10,000 rpm for 10 min, and the supernatant (designated as acid-treated prehydrolysate) was separated. The acid-treated prehydrolysate sample was analysed for the reduced sugar concentration and employed further to be used as feedstock for biohydrogen production by *E. cloacae* DT-1 strain.

#### Enzymatic saccharification of pretreated biofilm biomass

The pretreated biofilm biomass pellet was processed for enzymatic hydrolysis for its conversion to reducing sugars. The pretreated biofilm biomass sample was acidified with RO water, and the pH was brought down to 5 and enzymatic treatment was carried out at 50 °C for 24 h by adding cellulase enzyme. The hydrolysed sample was analysed to monitor the concentration of sugar before use as feedstock for dark fermentative biohydrogen production by *E. cloacae* DT-1.

#### Batch dark fermentation experiments

Initially, laboratory-scale studies were performed in 120-mL scale anaerobic serum bottles to optimize the biofilm prehydrolysate sugar concentration for maximum hydrogen production. For this, 10, 20, 30 and 40% (v/v) of acid-treated biofilm prehydrolysate was supplemented separately into the BSH medium as the sole feedstock. The initial pH of media was maintained at 7.5. The media were prepared anaerobically as mentioned previously [42]. 10% (v/v) of freshly grown DT-1 culture was used as inoculum. The bottles were incubated at 37 °C for 72 h under static conditions. The composition of biogas generated during the fermentation process was monitored by gas chromatography.

Laboratory-scale batch fermentative hydrogen production studies were conducted in 2000-mL serum bottles (batch reactors) containing 160 mL of anaerobically prepared BSH medium supplemented separately with acid-treated prehydrolysate (10% v/v, 4.3 g/L of reducing sugars) and enzymatically hydrolysed biofilm sugar (6 g/L reducing sugar) as feedstock. The initial pH of media was maintained at 7.5. The media were prepared anaerobically as mentioned previously [42, 44]. 10% (v/v) freshly grown DT-1 culture was used as inoculum. The bottles were incubated at 37 °C for 72 h under static conditions. Biogas generated during the fermentation process was connected with water displacement set up so as to displace the biogas as soon as it was produced inside the batch fermenter. Volumetric biogas production was monitored by measuring the displaced water collected in a graduated inverted water displacement system containing saline solution at ambient temperature. Qualitative detection of hydrogen production was carried out by gas chromatography.

#### Analytical methods

Bacterial growth was detected by measuring the optical density at 600 nm in a spectrophotometer. Biogas composition was generated in the headspace during the dark fermentation process Soluble metabolites was detected by gas chromatographic analysis (7890A, Agilent Technologies, USA) by following the protocols as reported earlier [44]. All the experiments were performed in duplicate. High-Performance Liquid Chromatograph (HPLC, Agilent 1100 series, USA) equipped with Sugar-PAK.1 column (Water Research, USA) was used for the detection of ethanol production. Water was used as the mobile phase at a flow rate of 0.6 mL/min. All the experiments were performed in duplicate. Sugar concentrations were measured using the 2,5-dinitro-salicylic acid method [81].

### Flocculation efficiency

Flocculation efficiency (FE) was calculated based on changes in OD_430_ and cell numbers of the uncaptured *Isochrysis* cells in the co-cultivation medium at time 0 and at the end of experiment according to the following formula: ${\text{FE}}\% = \frac{A - B}{A} \times 100\%$ where *A* = OD: the cell number at time 0; and *B* = OD: the cell number at the end of experiment.

### Sources of ROC

Raw ROC was collected from a reclamation facility of a local sewage treatment plant in which the biologically treated secondary effluent was treated by a UF (0.04 mm)-RO system to remove salts and other contaminants to produce recycled wastewater. The general characteristics of the ROC samples and secondary effluent used for this study are given in Additional file 12: Table S3. The collected samples were stored at −20 °C and elevated to room temperature before use.

### Statistical analysis

Most of the experiments in this study were conducted in triplicate. All data are expressed as a mean ± standard deviation. The experimental data were subjected to the one-way analysis of variance (ANOVA) as implemented in the GraphPad InStat 3 statistics platform. Tukey simultaneous tests were conducted to determine the statistical differences between treatments. To ascertain that the observed variations were statistically significant, the probability (*P*) values were determined. A 95% confidence level (*P* < 0.05) was applied for all analyses.

## Additional files

**Additional file 1: Figure S1.** Images of isolated saline biofilms. (A–O) Saline biofilms isolated from the saline lakes and marine habitats around Melbourne, Victoria (Australia). Microscopic analysis under UV light showed typical red fluorescence of chlorophyll molecules accumulated in filamentous and unicellular cyanobacterial and microalgal components of most of them (M as an example). (N) is an example of biofilm with microalgal components attached to the non-photosynthetic filaments. (M, N) Images under UV light; (O) stained for lipids with Nile Red. Scale bars: (A–C), 1 cm, (D–O), 20 µm.

**Additional file 2: Figure S2.** Spatial distribution of photosynthetic components within biofilms. (A, B) Diatoms growing within Biofilm #52; (C) BAPS-52-5 diatom (#4) isolated from Biofilm #52; (D) BAPS-52-4 diatom (#5) isolated from Biofilm #52; (E) Biofilm #21; (F) BAPS-21-1 green algae (#1) isolated from Biofilm #21. f1: BAPS-52-1 filaments; (B) image under UV light. Scale bars: (A, B, C, E), 20 µm; (D), 5 µm and (F), 10 µm.

**Additional file 3: Figure S3.** Biofilms growth patterns. (A) Biofilm #52 is floating on the surface of F2 medium (a) and attached to the glass walls (b, c). (B) Biofilms grown on agar plates. (h) image of Biofilm #52 under UV light. Scale bars: A (a, b, c),B (f),1 cm;B (a–e, g and h), 25 µm.

**Additional file 4: Figure S4.** A–F Characterization of components isolated from Biofilm #52 and Biofilm #21. A Characterization of BAPS-52-1. (A–E) Images of BAPS-52-1; Scale bars: (A, B, C), 20 µm; (D, E), 10 µm. (F) Phylogenetic tree. B: Characterization of BAPS-52-2. (A–E) Images of BAPS-52-1; Scale bars: (A, B, C, D), 20 µm; (E), 3 µm; (F) Phylogenetic tree. C: Characterization of BAPS-52-3. (A, B) Images of BAPS-52-3; Scale bars: (A, B), 20 µm; (C) Phylogenetic tree. D: Characterization of BAPS-52-4. (A, B) Images of BAPS-52-4; Scale bars: (A, B), 20 µm; (C) Phylogenetic tree. E: Characterization of BAPS-52-5. (A–F) Images of BAPS-52-5 diatom; Scale bars: (A, B), 20 µm; (C–F), 10 µm; (G) Phylogenetic tree. (D, F) staining for lipids with Nile Red. F: Characterization of BAPS-21-1. (A–D) Images of BAPS-21-1; Scale bars: (A–D), 20 µm; (E) Phylogenetic tree. Scale bars: 20 µM.

**Additional file 5: Figure S5.** Biofilm #52 growth in nutrient sufficient and nutrient-depleted media. (A) Biofilm #52 grown in nutrient sufficient F2 media (7 days after adding a fresh F2 media); (B) Biofilm #52 grown in nutrient depleted F2 media (14 days after adding a fresh F2 media). Scale bars: (A, B), 1 cm.

**Additional file 6: Figure S6.** Attachments of microalgal cells to BAPS-52-1 and BAPS-52-2 filaments. Secretion of EPS from BAPS-52-2 (A) and BAPS-52-1 (B); (C, D) Attachment of BAPS-21-1 to BAPS-52-1 filaments; (E) Attachment of BAPS-52-4 to BAPS-52-1 filaments; (F) Biofilm produced by mono-cultured diatoms BAPS-52-5; (G-I) Attachment of BAPS-52-4 diatom to BAPS-52-1 filaments; (J) Attachment of BAPS-52-5 diatom to BAPS-52-1 filaments; (K) Attachment of BAPS-52-5 diatom to BAPS-52-2 filaments. Secreted EPS is shown by the red arrow. Scale bars represent: (A -K), 20 µm.

**Additional file 7: Table S1.** Zeta potentials of Biofilms #52 and its microalgal, cyanobacterial and diatom inhabitants.

**Additional file 8: Figure S7.** Bio-flocculation of different microalgal strains by BAPS-52-1, BAPS-52-2, and BAPS-52-1 + BAPS-52-2. (A–D) *Isochrysis* sp. cells co-cultured with a mixture of BAPS-52-1 + BAPS-52-2; (E–J) *Isochrysis* sp. cells co-cultured with BAPS-52-1; (G–J) *Isochrysis* sp. cells attached to the biofilm produced by BAPS-52-1 attached to the microscopic slide; (K–N) *N. oculata* cells co-cultured with a mixture of BAPS-52-1 + BAPS-52-2; (O) *Nannochloris* sp. cells co-cultured with a mixture of BAPS-52-1 + BAPS-52-2; (P) *Nannochloris* sp. cells co-cultured with BAPS-52-2 filaments. (C, F, H, J, N) Images under UV light. Scale bars represent: (A–L, N–P), 50 µm; (M), 20 µm.

**Additional file 9: Table S1**. The chemical composition of SeSW.

**Additional file 10: Figure S8.** Mortality rates of *A. compressa* in untreated and treated by Biofilm #52 SeSW.

**Additional file 11: Figure S9.** Images of Biofilm #52 grown in SeSW. Biofilm #52 grown in nutrient depleted F2 media before the experiment. (B) Biofilm #52 grown 3 days in SeSW.

**Additional file 12: Table S3.** Chemical composition of ROC stream.

**Additional file 13: Table S4.** Chemical composition of ROC streams after treatment with assembled biofilm.

**Additional file 14: Table S5.** List of primers used for genotyping of biofilms components.

## Abbreviations

BAPS: biofilm-associated photosynthetic species
DW: dry weight
EPS: extracellular polymeric substance
FE: flocculation efficiency
GC: gas chromatography
OD: optical density
RO: reverse osmosis
ROC: reverse osmosis concentrate
SeSW: selenium-rich synthetic wastewater
