# Supplementary figures and images for: Applications of microalgal biofilms for wastewater treatment and bioenergy production
Source: Biotechnol Biofuels. 2017 May 10;10:120. doi: 10.1186/s13068-017-0798-9 (PMC5424312; doi:10.1186/s13068-017-0798-9)

## Slide 1
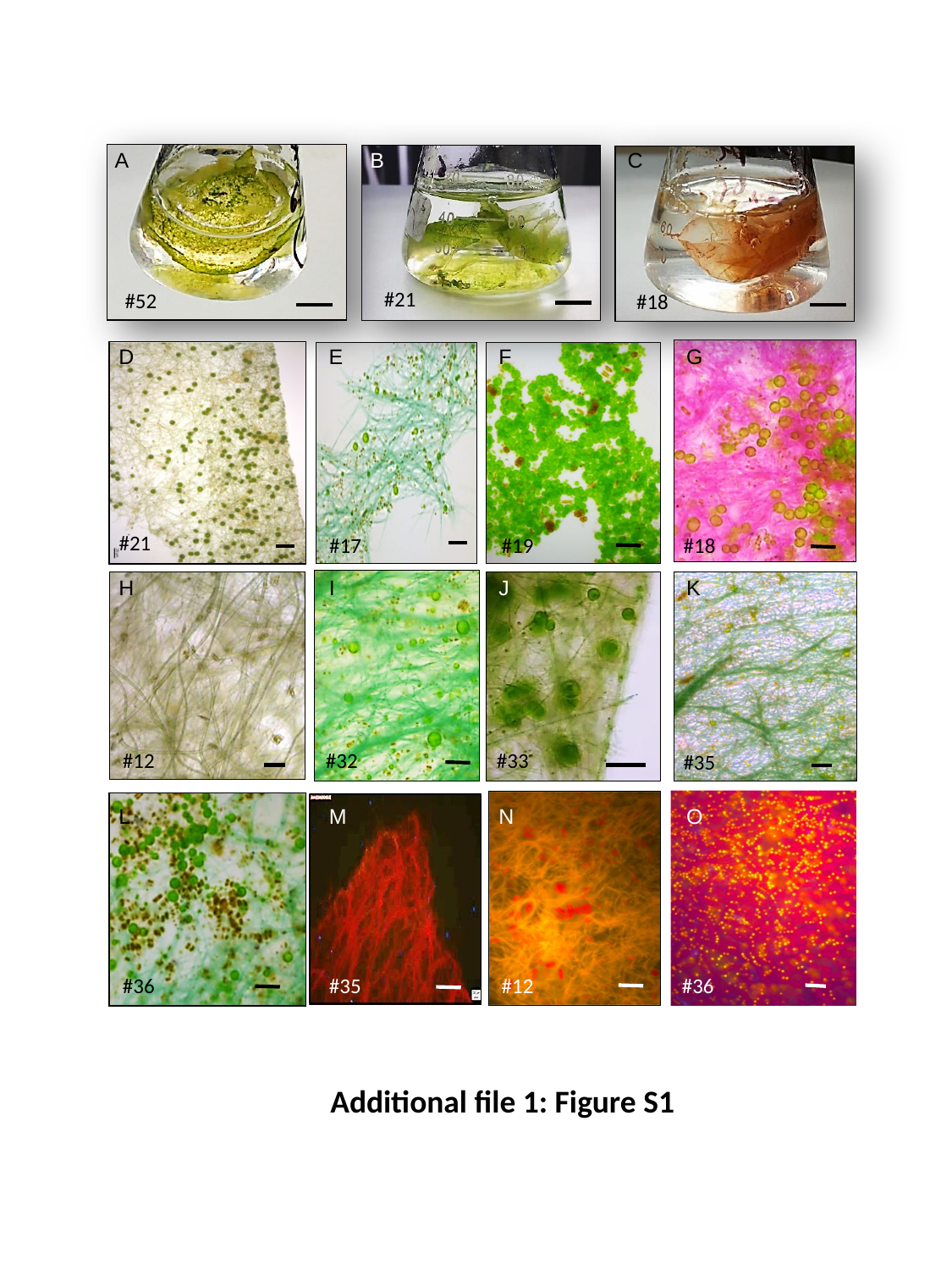

A
B
C
#21
#52
#18
D
E
F
G
#21
#17
#19
#18
H
I
J
K
#33
#32
#12
#35
L
M
N
O
#36
#12
#35
#36
Additional file 1: Figure S1

Supplement: Supplementary file 1 — Additional file 1: Figure S1. Images of isolated saline biofilms. (A–O) Saline biofilms isolated from the saline lakes and marine habitats around Melbourne, Victoria (Australia). Microscopic analysis under UV light showed typical red fluorescence of chlorophyll molecules accumulated in filamentous and unicellular cyanobacterial and microalgal components of most of them (M as an example). (N) is an example of biofilm with microalgal components attached to the non-photosynthetic filaments. (M, N) Images under UV light; (O) stained for lipids with Nile Red. Scale bars: (A–C), 1 cm, (D–O), 20 µm. [file 13068_2017_798_MOESM1_ESM.pptx]

## Slide 1
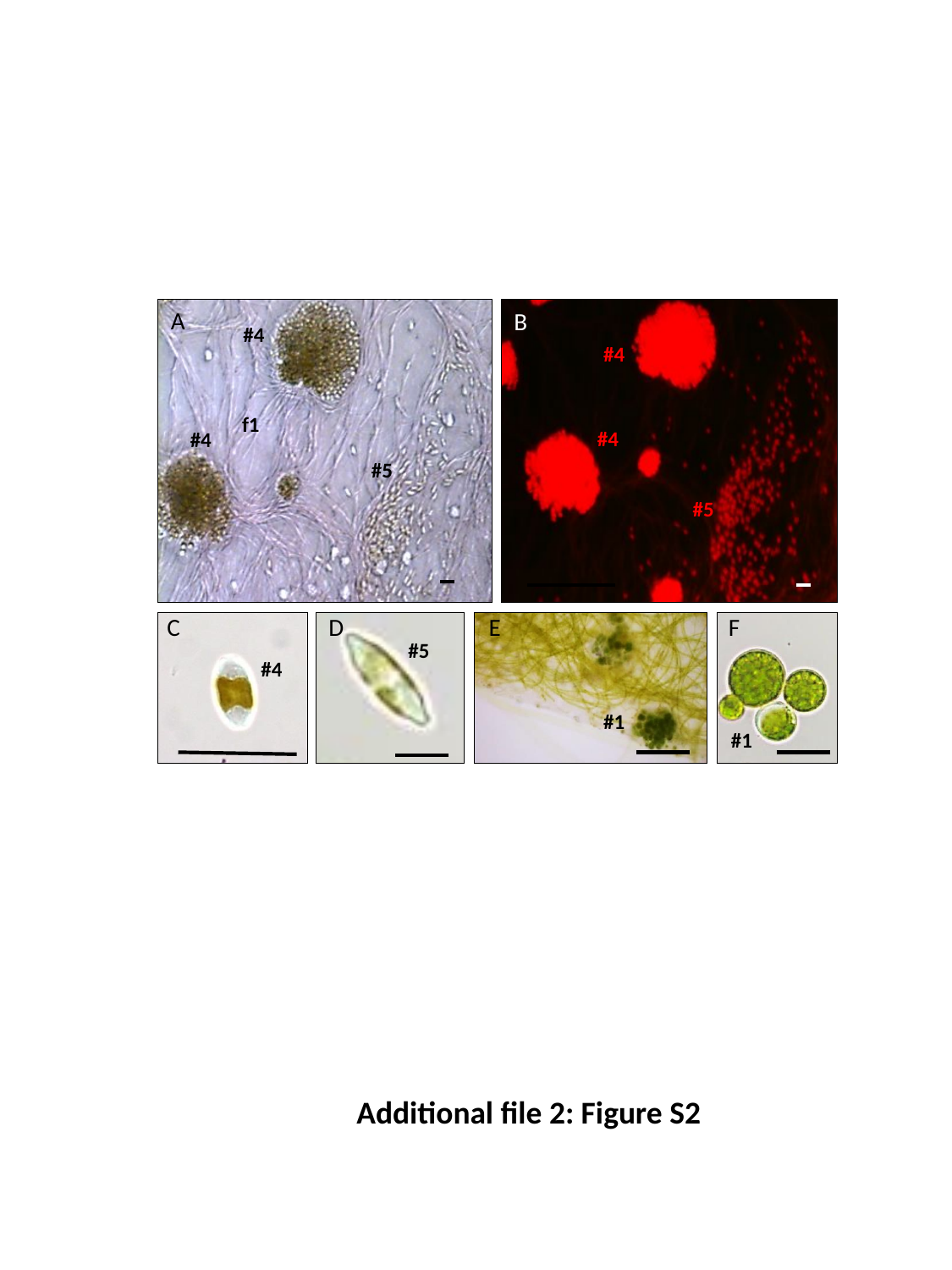

A
B
#4
#4
f1
#4
#4
#5
#5
C
D
E
F
#5
#4
#1
#1
Additional file 2: Figure S2

Supplement: Supplementary file 2 — Additional file 2: Figure S2. Spatial distribution of photosynthetic components within biofilms. (A, B) Diatoms growing within Biofilm #52; (C) BAPS-52-5 diatom (#4) isolated from Biofilm #52; (D) BAPS-52-4 diatom (#5) isolated from Biofilm #52; (E) Biofilm #21; (F) BAPS-21-1 green algae (#1) isolated from Biofilm #21. f1: BAPS-52-1 filaments; (B) image under UV light. Scale bars: (A, B, C, E), 20 µm; (D), 5 µm and (F), 10 µm. [file 13068_2017_798_MOESM2_ESM.pptx]

## Slide 1
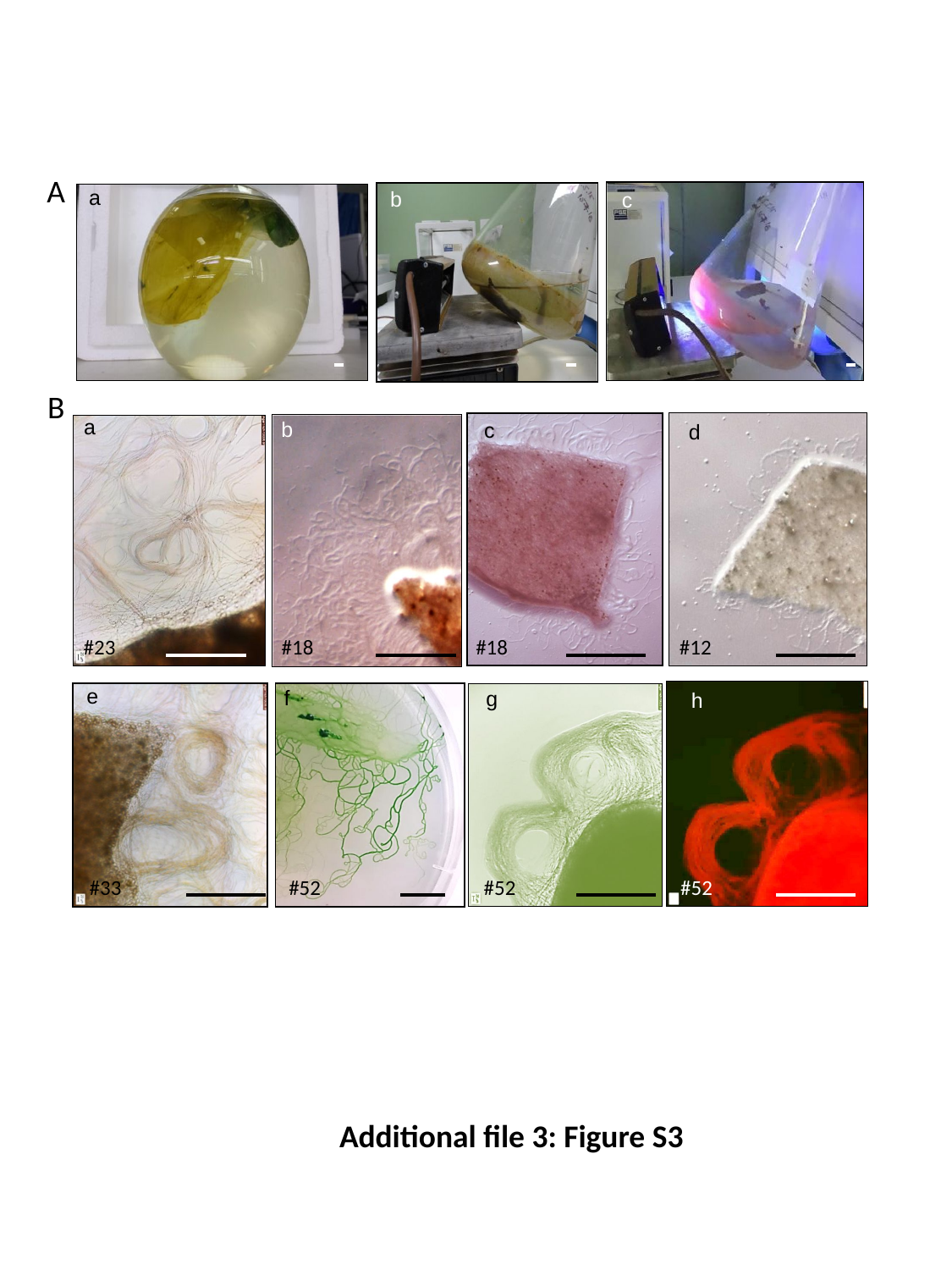

A
a
b
c
B
a
b
c
d
#12
#23
#18
#18
e
f
g
h
#52
#33
#52
#52
Additional file 3: Figure S3

Supplement: Supplementary file 3 — Additional file 3: Figure S3. Biofilms growth patterns. (A) Biofilm #52 is floating on the surface of F2 medium (a) and attached to the glass walls (b, c). (B) Biofilms grown on agar plates. (h) image of Biofilm #52 under UV light. Scale bars: A (a, b, c),B (f),1 cm;B (a–e, g and h), 25 µm. [file 13068_2017_798_MOESM3_ESM.pptx]

## Slide 1
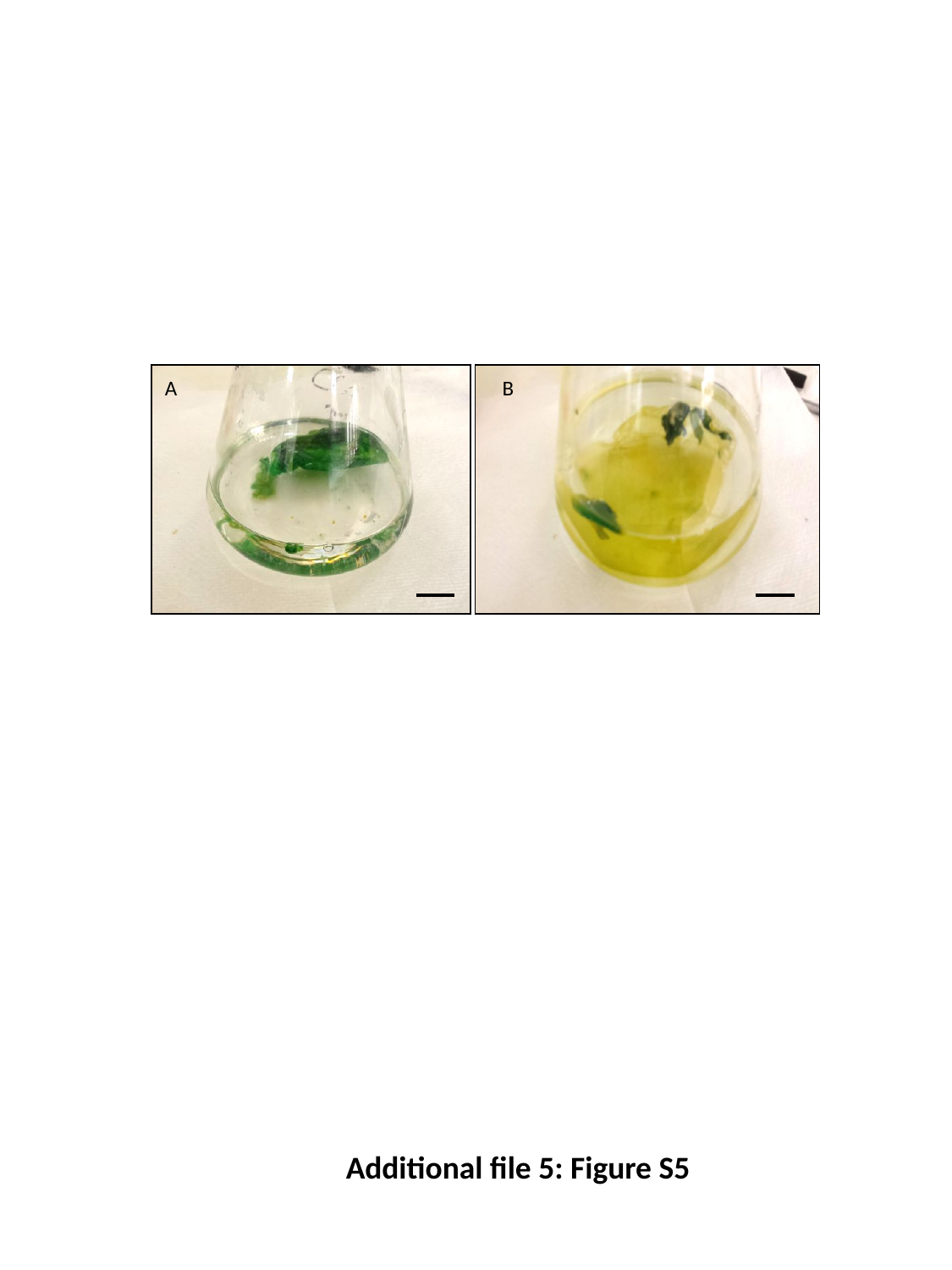

A
B
Additional file 5: Figure S5

Supplement: Supplementary file 5 — Additional file 5: Figure S5. Biofilm #52 growth in nutrient sufficient and nutrient-depleted media. (A) Biofilm #52 grown in nutrient sufficient F2 media (7 days after adding a fresh F2 media); (B) Biofilm #52 grown in nutrient depleted F2 media (14 days after adding a fresh F2 media). Scale bars: (A, B), 1 cm. [file 13068_2017_798_MOESM5_ESM.pptx]

## Slide 1
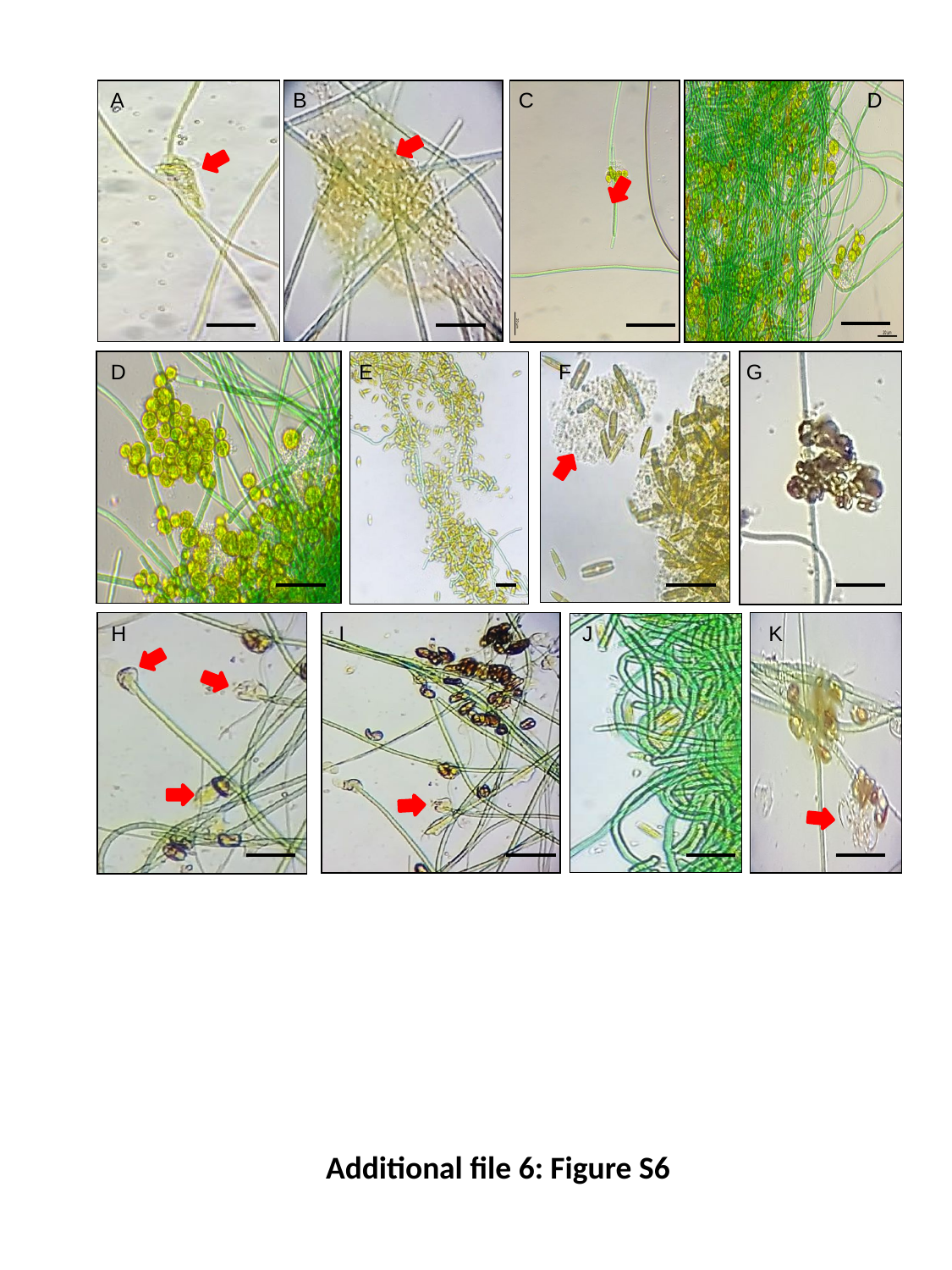

A
B
C
D
D
E
F
G
H
I
J
K
Additional file 6: Figure S6

Supplement: Supplementary file 6 — Additional file 6: Figure S6. Attachments of microalgal cells to BAPS-52-1 and BAPS-52-2 filaments. Secretion of EPS from BAPS-52-2 (A) and BAPS-52-1 (B); (C, D) Attachment of BAPS-21-1 to BAPS-52-1 filaments; (E) Attachment of BAPS-52-4 to BAPS-52-1 filaments; (F) Biofilm produced by mono-cultured diatoms BAPS-52-5; (G-I) Attachment of BAPS-52-4 diatom to BAPS-52-1 filaments; (J) Attachment of BAPS-52-5 diatom to BAPS-52-1 filaments; (K) Attachment of BAPS-52-5 diatom to BAPS-52-2 filaments. Secreted EPS is shown by the red arrow. Scale bars represent: (A -K), 20 µm. [file 13068_2017_798_MOESM6_ESM.pptx]

## Slide 1
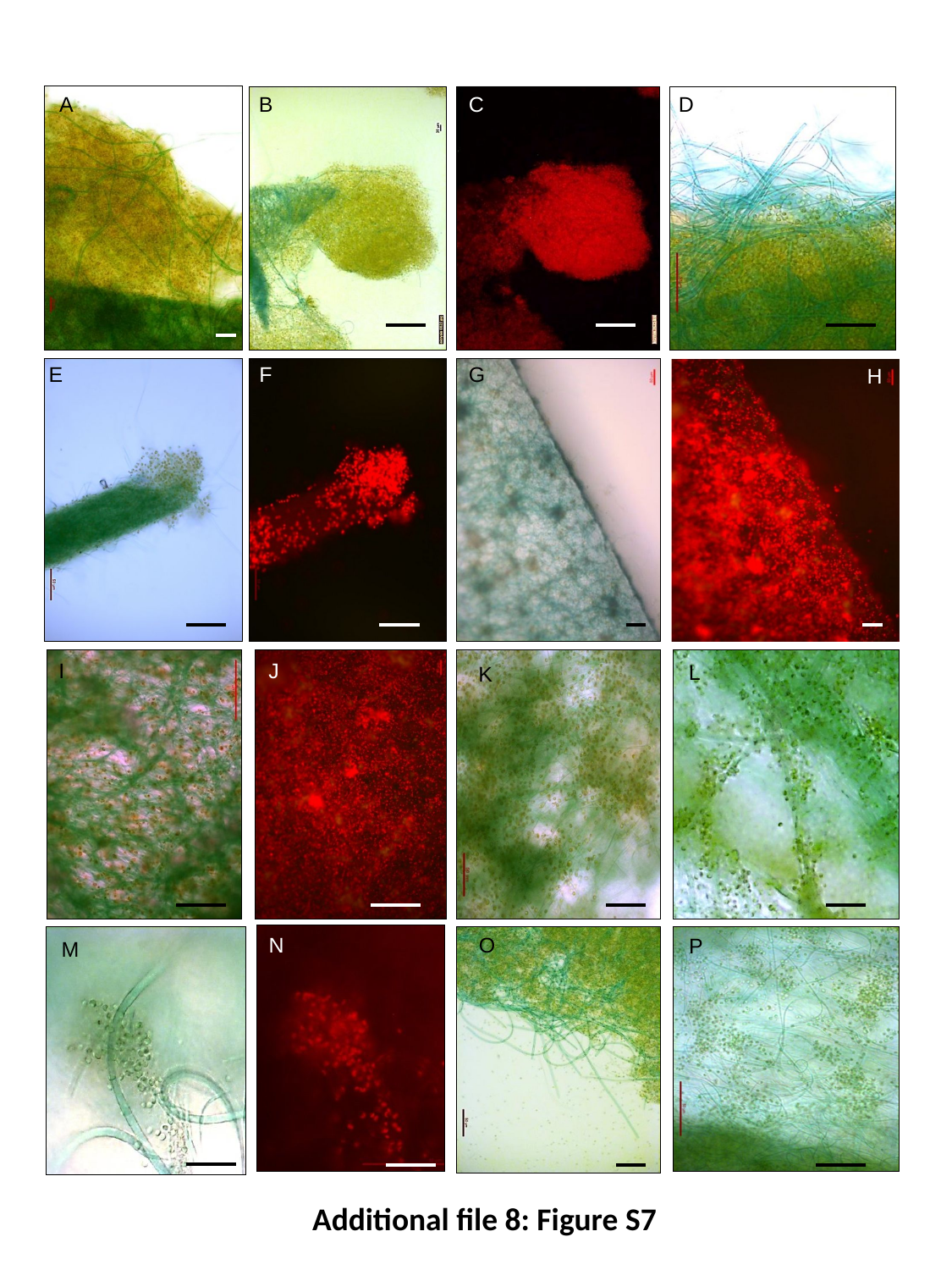

A
B
C
D
E
F
G
H
52-1
I
J
L
K
M
N
O
P
M
20µm
Additional file 8: Figure S7

Supplement: Supplementary file 8 — Additional file 8: Figure S7. Bio-flocculation of different microalgal strains by BAPS-52-1, BAPS-52-2, and BAPS-52-1 + BAPS-52-2. (A–D) Isochrysis sp. cells co-cultured with a mixture of BAPS-52-1 + BAPS-52-2; (E–J) Isochrysis sp. cells co-cultured with BAPS-52-1; (G–J) Isochrysis sp. cells attached to the biofilm produced by BAPS-52-1 attached to the microscopic slide; (K–N) N. oculata cells co-cultured with a mixture of BAPS-52-1 + BAPS-52-2; (O) Nannochloris sp. cells co-cultured with a mixture of BAPS-52-1 + BAPS-52-2; (P) Nannochloris sp. cells co-cultured with BAPS-52-2 filaments. (C, F, H, J, N) Images under UV light. Scale bars represent: (A–L, N–P), 50 µm; (M), 20 µm. [file 13068_2017_798_MOESM8_ESM.pptx]

## Slide 1
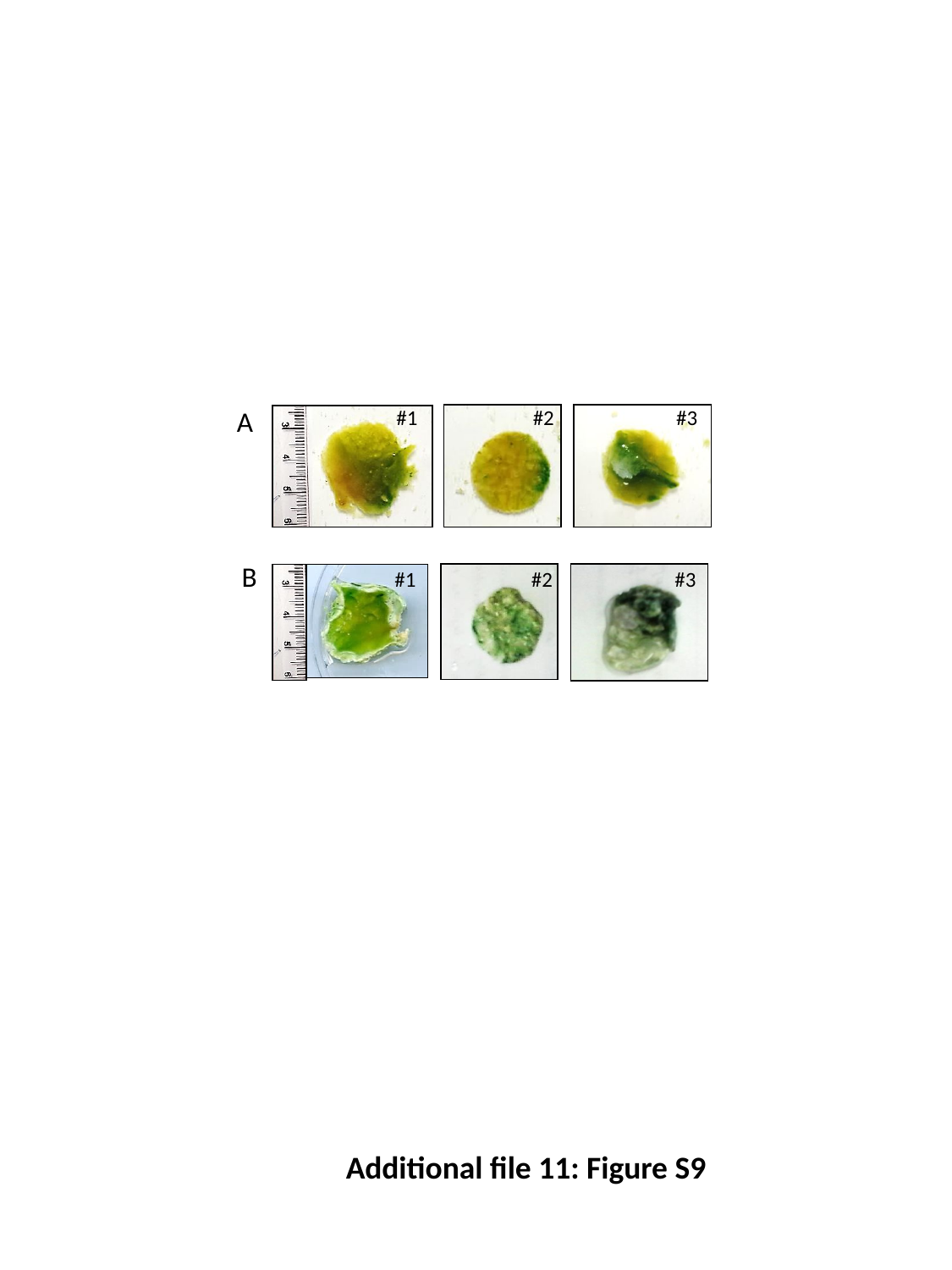

A
#1
#2
#3
B
#1
#2
#3
Additional file 11: Figure S9

Supplement: Supplementary file 11 — Additional file 11: Figure S9. Images of Biofilm #52 grown in SeSW. Biofilm #52 grown in nutrient depleted F2 media before the experiment. (B) Biofilm #52 grown 3 days in SeSW. [file 13068_2017_798_MOESM11_ESM.pptx]
