# Supplementary material for: Applications of microalgal biofilms for wastewater treatment and bioenergy production
Source: Biotechnol Biofuels. 2017 May 10;10:120. doi: 10.1186/s13068-017-0798-9 (PMC5424312; doi:10.1186/s13068-017-0798-9)
Supplement: Supplementary file 4 — Additional file 4: Figure S4. A–F Characterization of components isolated from Biofilm #52 and Biofilm #21. A Characterization of BAPS-52-1. (A–E) Images of BAPS-52-1; Scale bars: (A, B, C), 20 µm; (D, E), 10 µm. (F) Phylogenetic tree. B: Characterization of BAPS-52-2. (A–E) Images of BAPS-52-1; Scale bars: (A, B, C, D), 20 µm; (E), 3 µm; (F) Phylogenetic tree. C: Characterization of BAPS-52-3. (A, B) Images of BAPS-52-3; Scale bars: (A, B), 20 µm; (C) Phylogenetic tree. D: Characterization of BAPS-52-4. (A, B) Images of BAPS-52-4; Scale bars: (A, B), 20 µm; (C) Phylogenetic tree. E: Characterization of BAPS-52-5. (A–F) Images of BAPS-52-5 diatom; Scale bars: (A, B), 20 µm; (C–F), 10 µm; (G) Phylogenetic tree. (D, F) staining for lipids with Nile Red. F: Characterization of BAPS-21-1. (A–D) Images of BAPS-21-1; Scale bars: (A–D), 20 µm; (E) Phylogenetic tree. Scale bars: 20 µM. [file 13068_2017_798_MOESM4_ESM.pptx]

## Slide 1
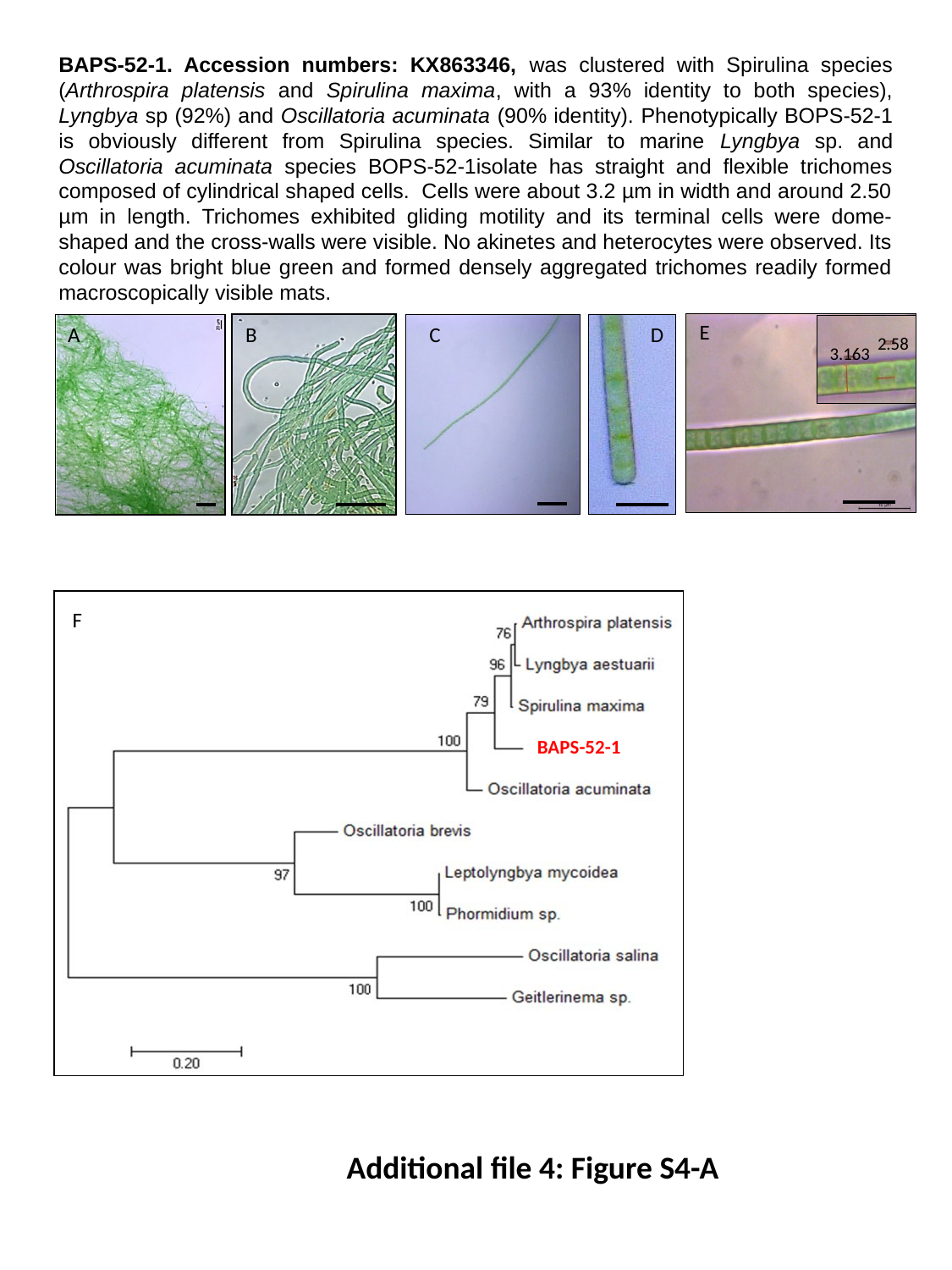

BAPS-52-1. Accession numbers: KX863346, was clustered with Spirulina species (Arthrospira platensis and Spirulina maxima, with a 93% identity to both species), Lyngbya sp (92%) and Oscillatoria acuminata (90% identity). Phenotypically BOPS-52-1 is obviously different from Spirulina species. Similar to marine Lyngbya sp. and Oscillatoria acuminata species BOPS-52-1isolate has straight and flexible trichomes composed of cylindrical shaped cells. Cells were about 3.2 µm in width and around 2.50 µm in length. Trichomes exhibited gliding motility and its terminal cells were dome-shaped and the cross-walls were visible. No akinetes and heterocytes were observed. Its colour was bright blue green and formed densely aggregated trichomes readily formed macroscopically visible mats.
E
A
B
C
C
D
2.58
3.163
F
BAPS-52-1
Additional file 4: Figure S4-A

## Slide 2
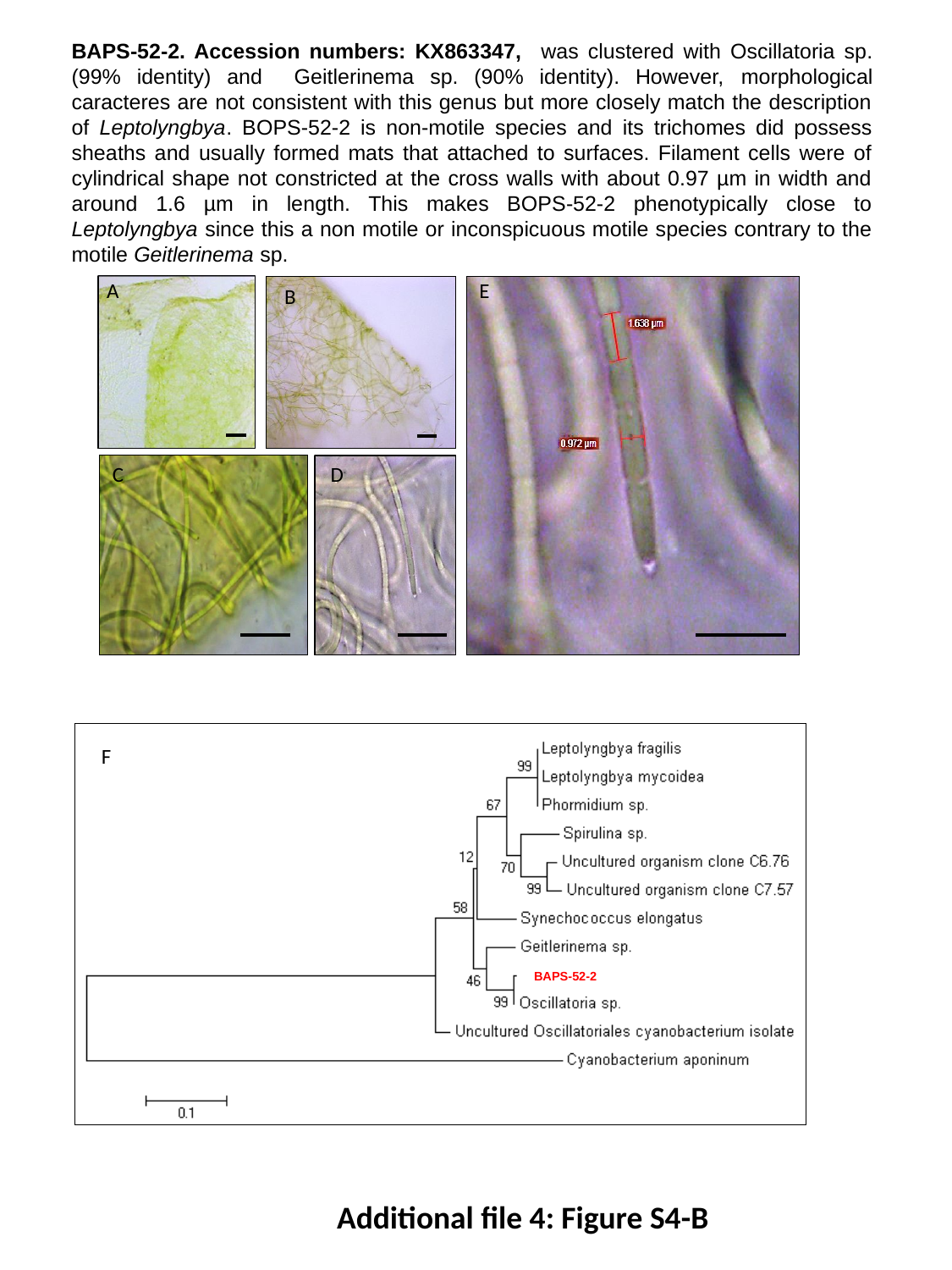

BAPS-52-2. Accession numbers: KX863347, was clustered with Oscillatoria sp. (99% identity) and Geitlerinema sp. (90% identity). However, morphological caracteres are not consistent with this genus but more closely match the description of Leptolyngbya. BOPS-52-2 is non-motile species and its trichomes did possess sheaths and usually formed mats that attached to surfaces. Filament cells were of cylindrical shape not constricted at the cross walls with about 0.97 µm in width and around 1.6 µm in length. This makes BOPS-52-2 phenotypically close to Leptolyngbya since this a non motile or inconspicuous motile species contrary to the motile Geitlerinema sp.
A
B
E
B
C
D
F
BAPS-52-2
Additional file 4: Figure S4-B

## Slide 3
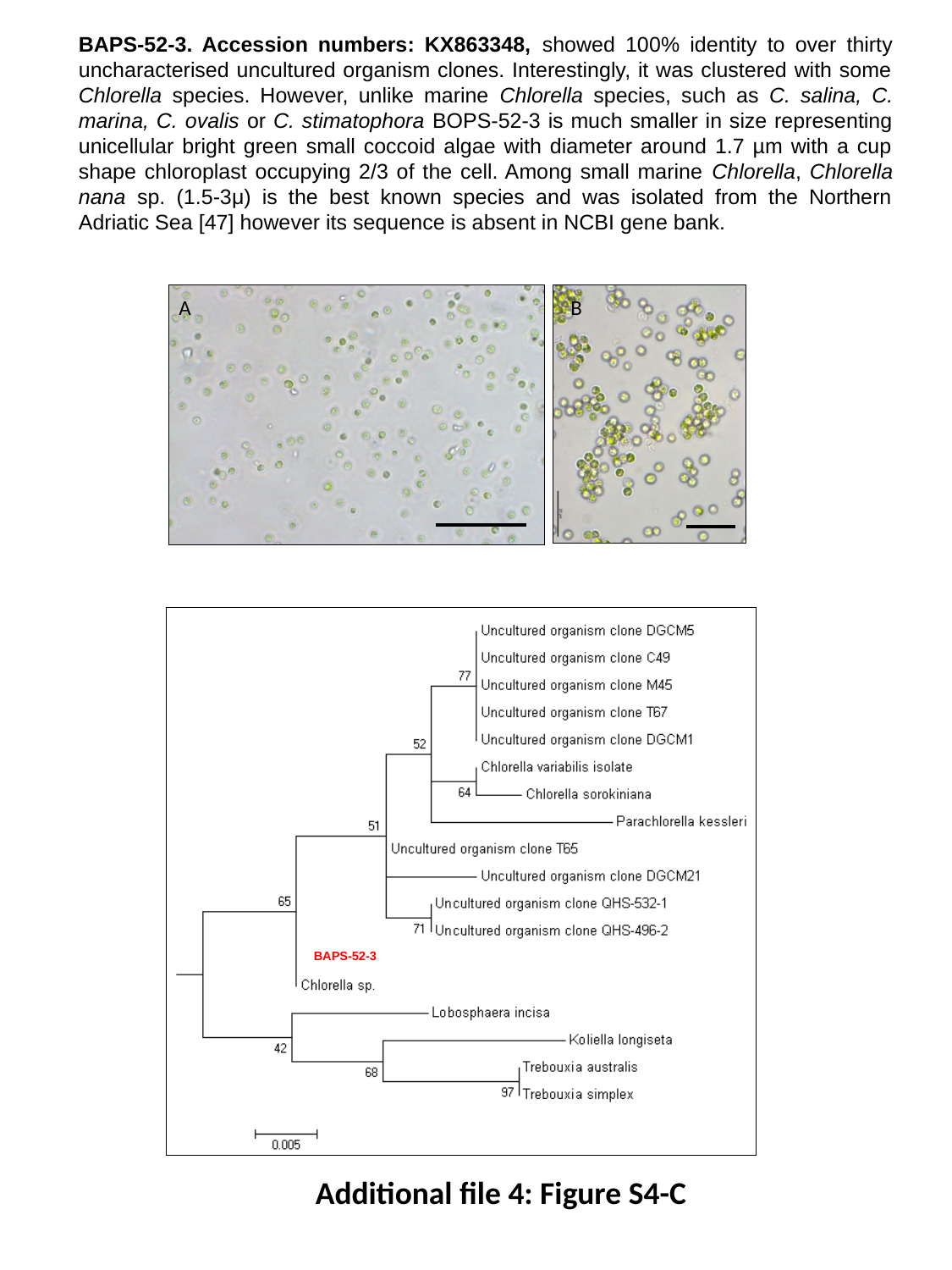

BAPS-52-3. Accession numbers: KX863348, showed 100% identity to over thirty uncharacterised uncultured organism clones. Interestingly, it was clustered with some Chlorella species. However, unlike marine Chlorella species, such as C. salina, C. marina, C. ovalis or C. stimatophora BOPS-52-3 is much smaller in size representing unicellular bright green small coccoid algae with diameter around 1.7 µm with a cup shape chloroplast occupying 2/3 of the cell. Among small marine Chlorella, Chlorella nana sp. (1.5-3μ) is the best known species and was isolated from the Northern Adriatic Sea [47] however its sequence is absent in NCBI gene bank.
A
B
C
BAPS-52-3
C
Additional file 4: Figure S4-C

## Slide 4
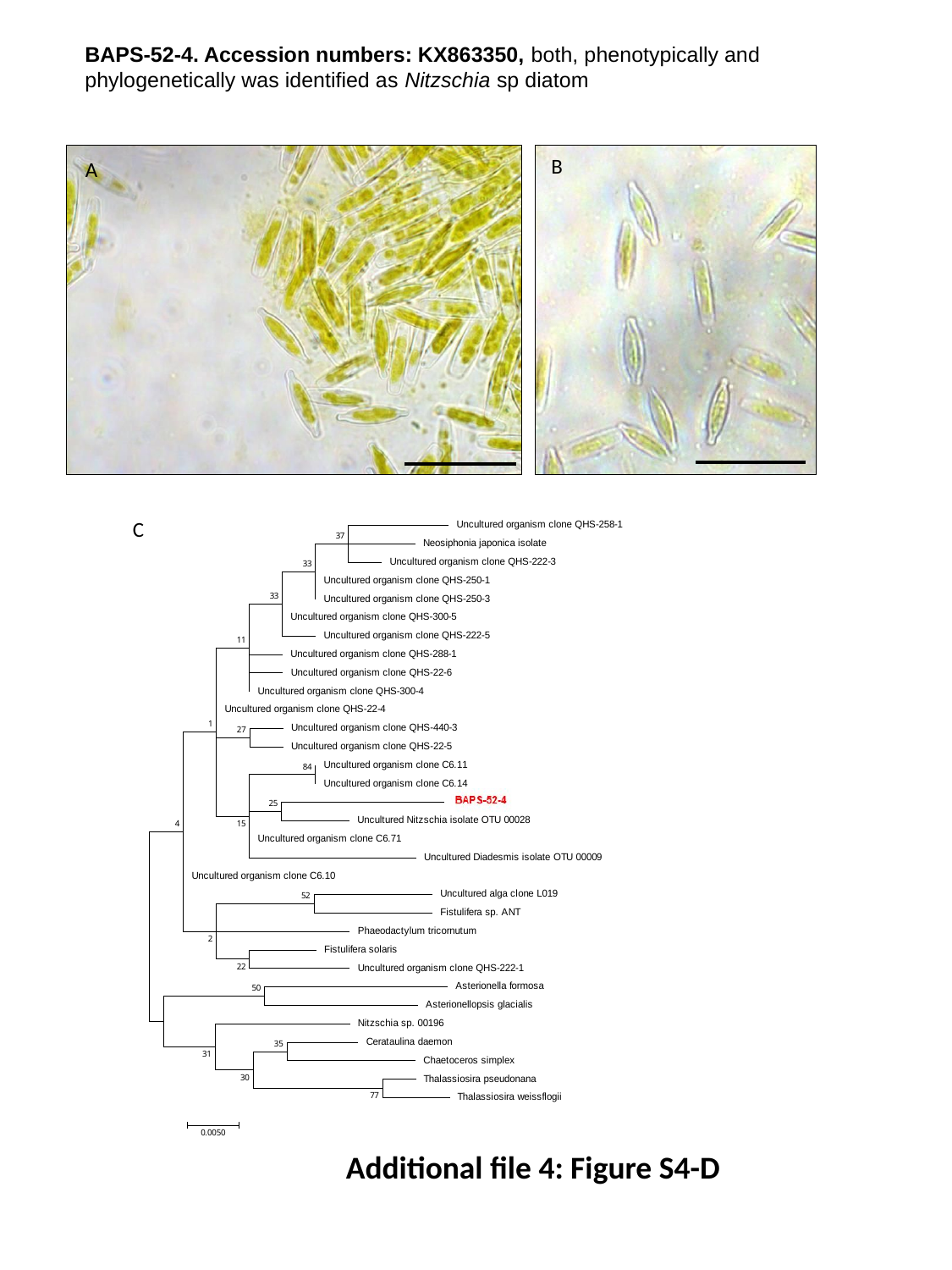

BAPS-52-4. Accession numbers: KX863350, both, phenotypically and phylogenetically was identified as Nitzschia sp diatom
A
B
A
C
Additional file 4: Figure S4-D

## Slide 5
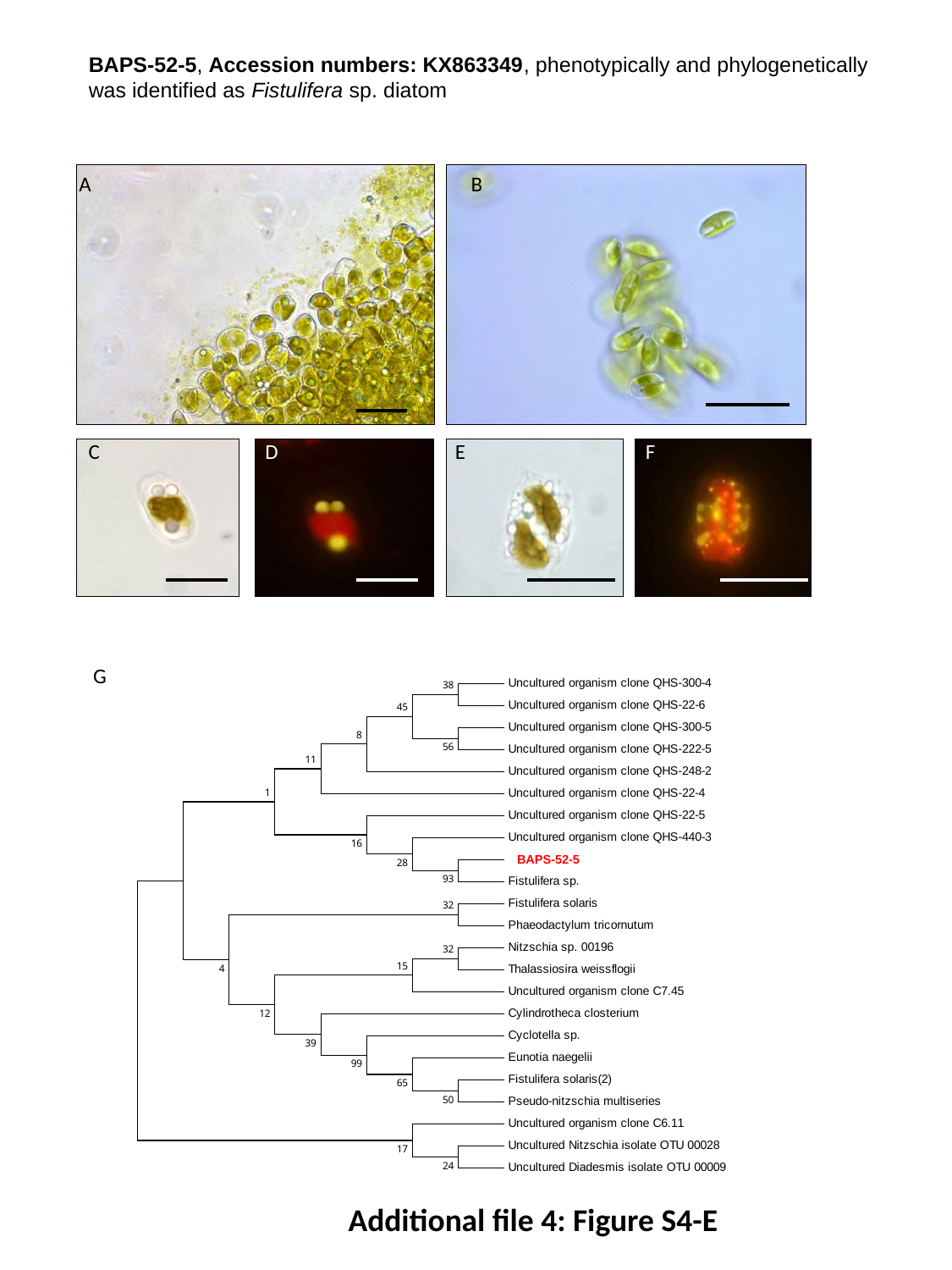

BAPS-52-5, Accession numbers: KX863349, phenotypically and phylogenetically was identified as Fistulifera sp. diatom
A
B
C
D
E
F
G
BAPS-52-5
Additional file 4: Figure S4-E

## Slide 6
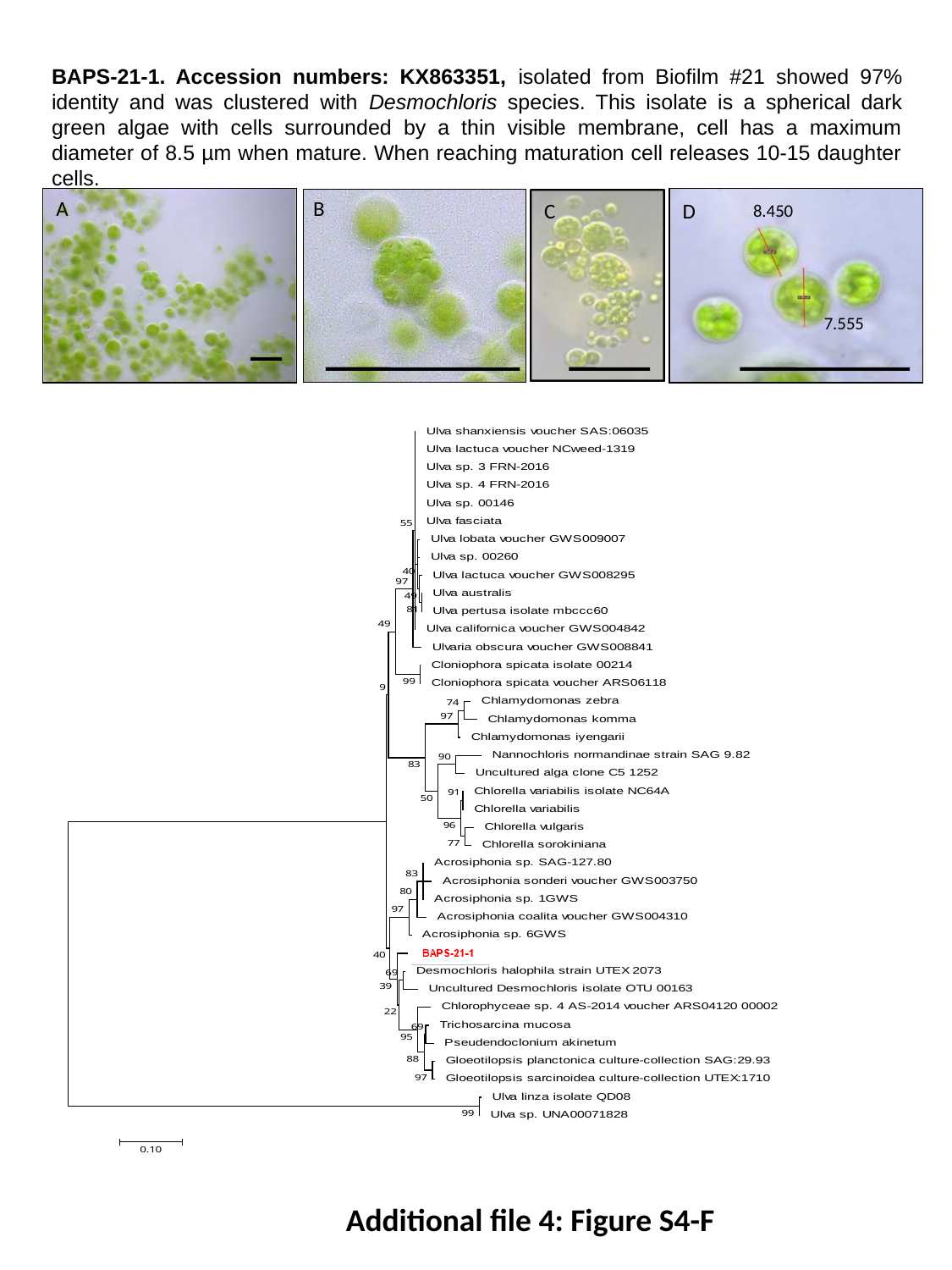

BAPS-21-1. Accession numbers: KX863351, isolated from Biofilm #21 showed 97% identity and was clustered with Desmochloris species. This isolate is a spherical dark green algae with cells surrounded by a thin visible membrane, cell has a maximum diameter of 8.5 µm when mature. When reaching maturation cell releases 10-15 daughter cells.
A
B
C
D
8.450
7.555
Additional file 4: Figure S4-F
