# Supplementary material for: Applications of microalgal biofilms for wastewater treatment and bioenergy production
Source: Biotechnol Biofuels. 2017 May 10;10:120. doi: 10.1186/s13068-017-0798-9 (PMC5424312; doi:10.1186/s13068-017-0798-9)
Supplement: Supplementary file 7 — Additional file 7: Table S1. Zeta potentials of Biofilms #52 and its microalgal, cyanobacterial and diatom inhabitants. [file 13068_2017_798_MOESM7_ESM.pptx]

## Slide 1
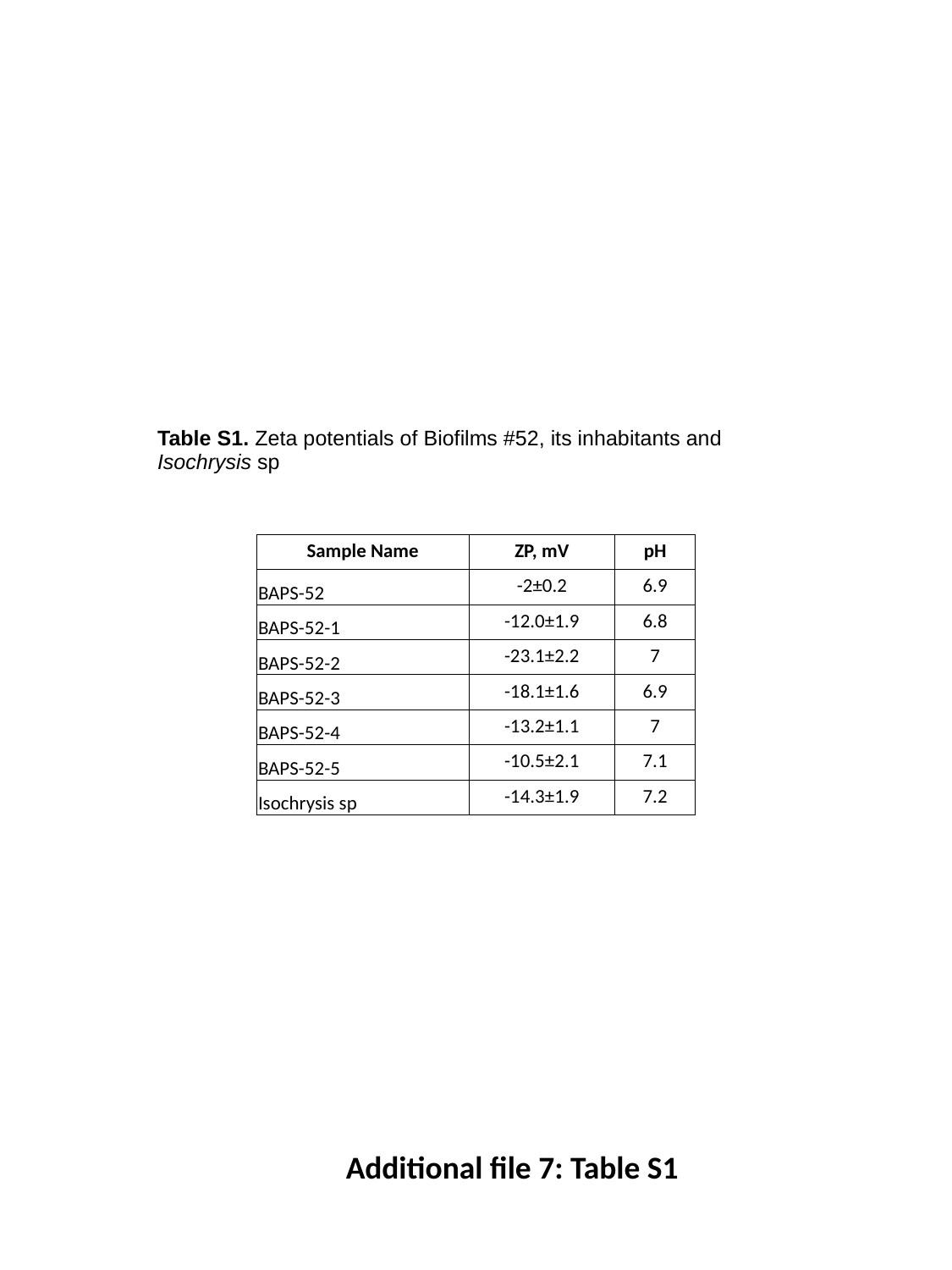

| Table S1. Zeta potentials of Biofilms #52, its inhabitants and Isochrysis sp | | | | |
| --- | --- | --- | --- | --- |
| | | | | |
| | Sample Name | ZP, mV | pH | |
| | BAPS-52 | -2±0.2 | 6.9 | |
| | BAPS-52-1 | -12.0±1.9 | 6.8 | |
| | BAPS-52-2 | -23.1±2.2 | 7 | |
| | BAPS-52-3 | -18.1±1.6 | 6.9 | |
| | BAPS-52-4 | -13.2±1.1 | 7 | |
| | BAPS-52-5 | -10.5±2.1 | 7.1 | |
| | Isochrysis sp | -14.3±1.9 | 7.2 | |
Additional file 7: Table S1
