# Supplementary material for: Applications of microalgal biofilms for wastewater treatment and bioenergy production
Source: Biotechnol Biofuels. 2017 May 10;10:120. doi: 10.1186/s13068-017-0798-9 (PMC5424312; doi:10.1186/s13068-017-0798-9)
Supplement: Supplementary file 9 — Additional file 9: Table S1. The chemical composition of SeSW. [file 13068_2017_798_MOESM9_ESM.pptx]

## Slide 1
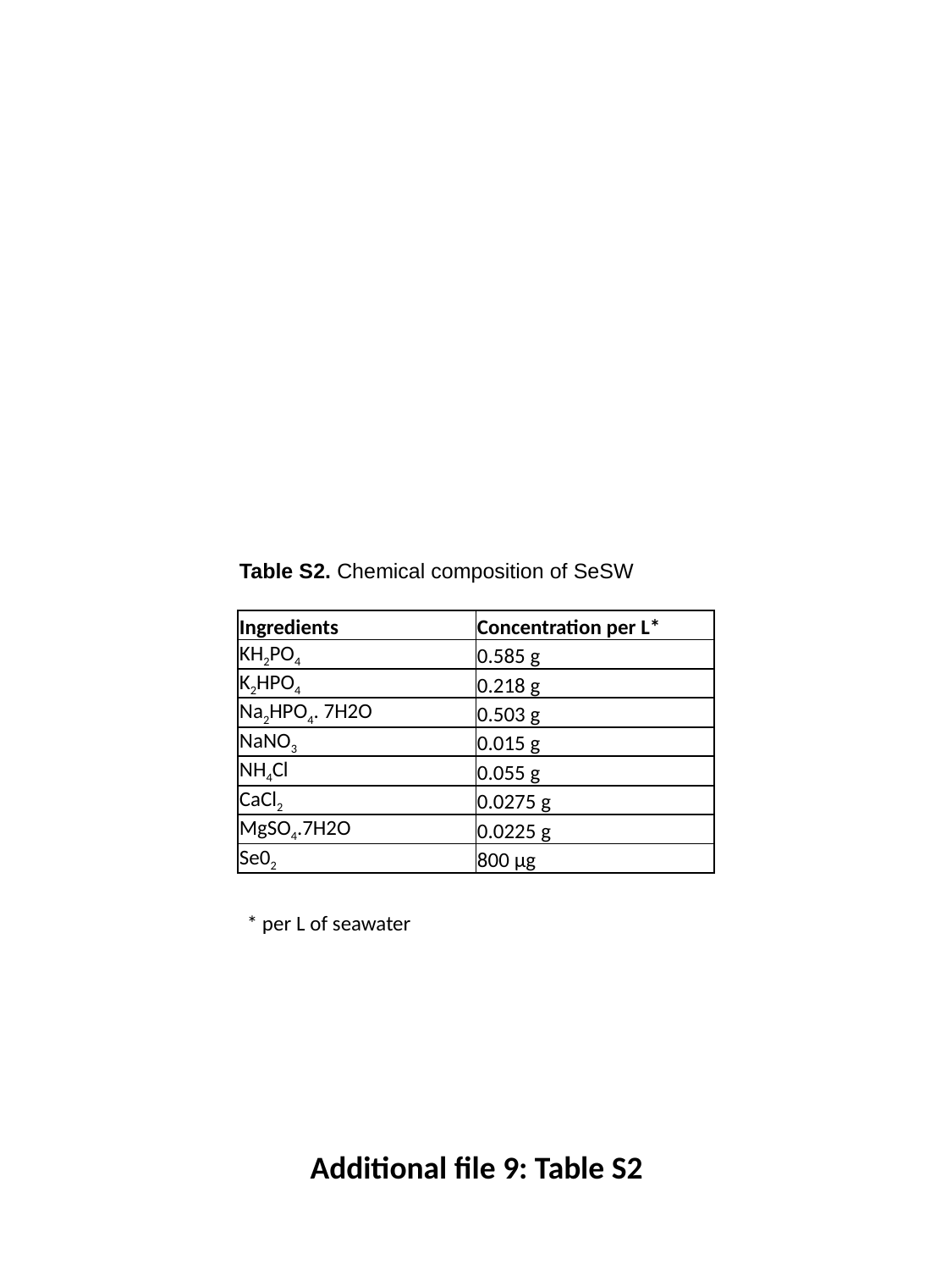

| Table S2. Chemical composition of SeSW | |
| --- | --- |
| | |
| Ingredients | Concentration per L\* |
| KH2PO4 | 0.585 g |
| K2HPO4 | 0.218 g |
| Na2HPO4. 7H2O | 0.503 g |
| NaNO3 | 0.015 g |
| NH4Cl | 0.055 g |
| CaCl2 | 0.0275 g |
| MgSO4.7H2O | 0.0225 g |
| Se02 | 800 µg |
* per L of seawater
Additional file 9: Table S2
