# Supplementary material for: Applications of microalgal biofilms for wastewater treatment and bioenergy production
Source: Biotechnol Biofuels. 2017 May 10;10:120. doi: 10.1186/s13068-017-0798-9 (PMC5424312; doi:10.1186/s13068-017-0798-9)
Supplement: Supplementary file 10 — Additional file 10: Figure S8. Mortality rates of A. compressa in untreated and treated by Biofilm #52 SeSW. [file 13068_2017_798_MOESM10_ESM.pptx]

## Slide 1
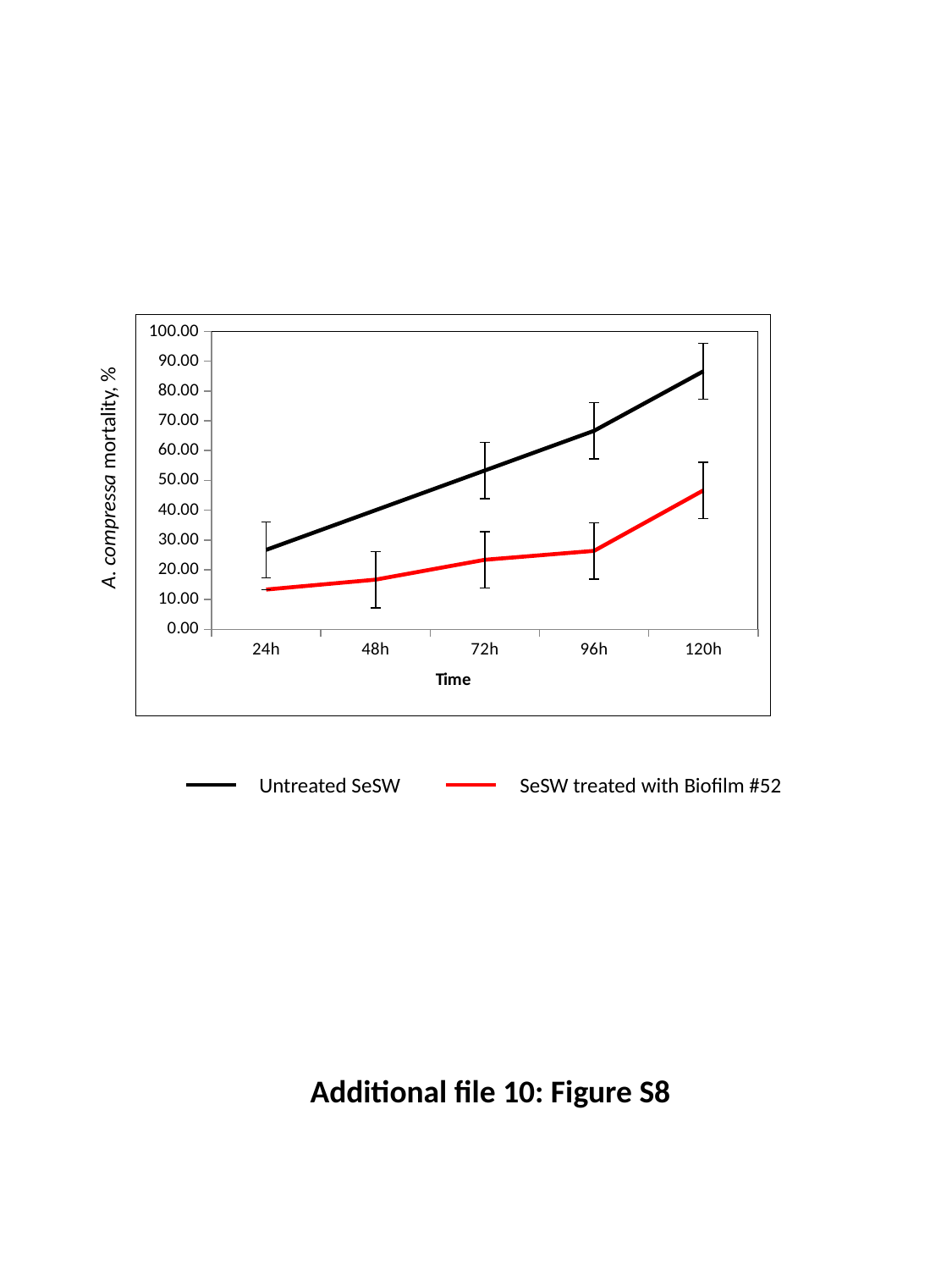

### Chart
| Category | Untreated SeWW | Biofilm 2 |
|---|---|---|
| 24h | 26.6666666666667 | 13.333333333333334 |
| 48h | 40.00000000000001 | 16.666666666666668 |
| 72h | 53.333333333333336 | 23.333333333333325 |
| 96h | 66.66666666666666 | 26.333333333333286 |
| 120h | 86.66666666666667 | 46.666666666666636 |A. compressa mortality, %
SeSW treated with Biofilm #52
Untreated SeSW
Additional file 10: Figure S8
