# Supplementary material for: Applications of microalgal biofilms for wastewater treatment and bioenergy production
Source: Biotechnol Biofuels. 2017 May 10;10:120. doi: 10.1186/s13068-017-0798-9 (PMC5424312; doi:10.1186/s13068-017-0798-9)
Supplement: Supplementary file 12 — Additional file 12: Table S3. Chemical composition of ROC stream. [file 13068_2017_798_MOESM12_ESM.pptx]

## Slide 1
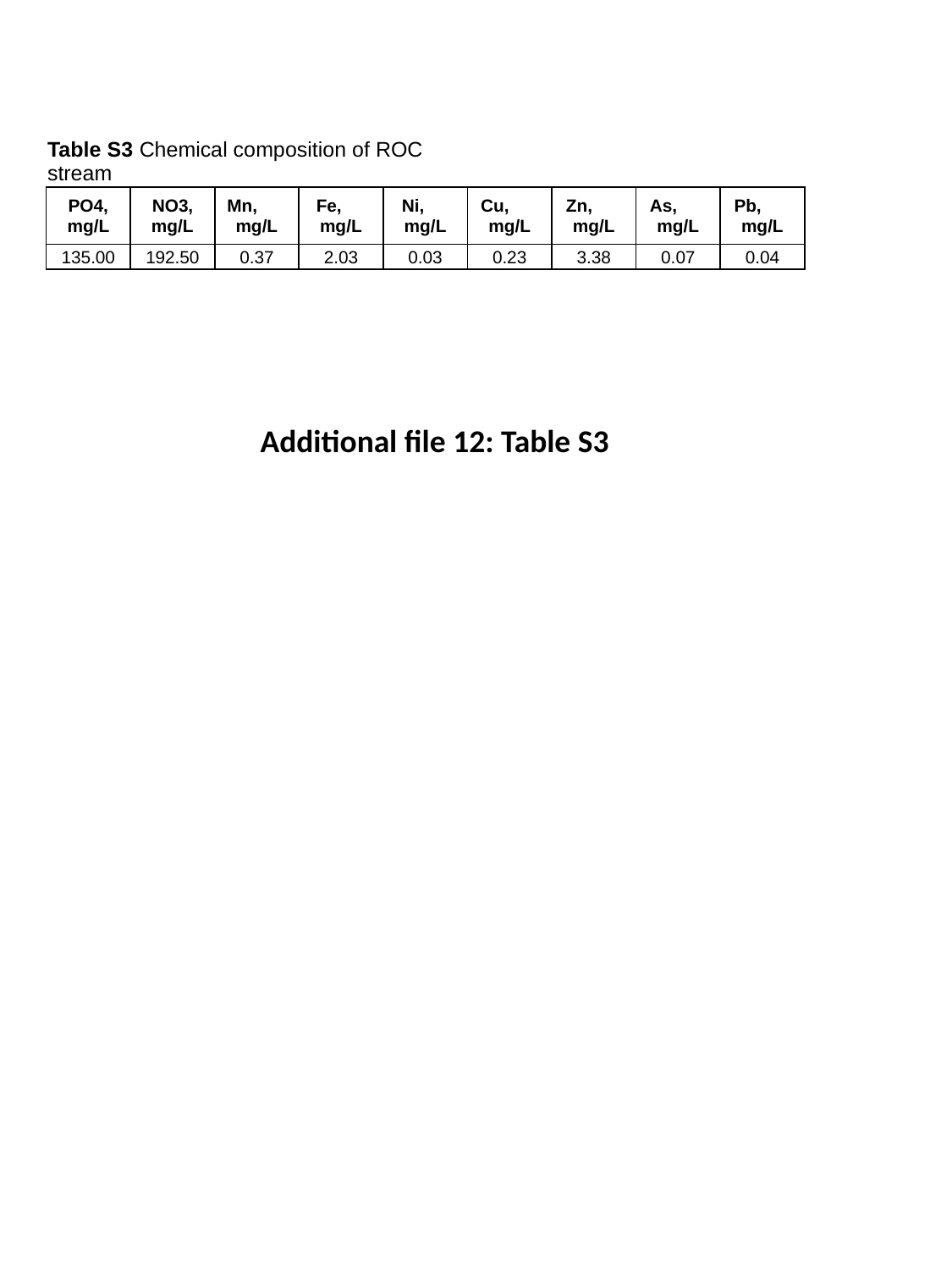

| Table S3 Chemical composition of ROC stream | | | | | | | | | |
| --- | --- | --- | --- | --- | --- | --- | --- | --- | --- |
| PO4, mg/L | NO3, mg/L | Mn, mg/L | Fe, mg/L | Ni, mg/L | Cu, mg/L | Zn, mg/L | As, mg/L | Pb, mg/L | |
| 135.00 | 192.50 | 0.37 | 2.03 | 0.03 | 0.23 | 3.38 | 0.07 | 0.04 | |
| | | | | | | | | | |
Additional file 12: Table S3
