# Supplementary material for: Applications of microalgal biofilms for wastewater treatment and bioenergy production
Source: Biotechnol Biofuels. 2017 May 10;10:120. doi: 10.1186/s13068-017-0798-9 (PMC5424312; doi:10.1186/s13068-017-0798-9)
Supplement: Supplementary file 13 — Additional file 13: Table S4. Chemical composition of ROC streams after treatment with assembled biofilm. [file 13068_2017_798_MOESM13_ESM.pptx]

## Slide 1
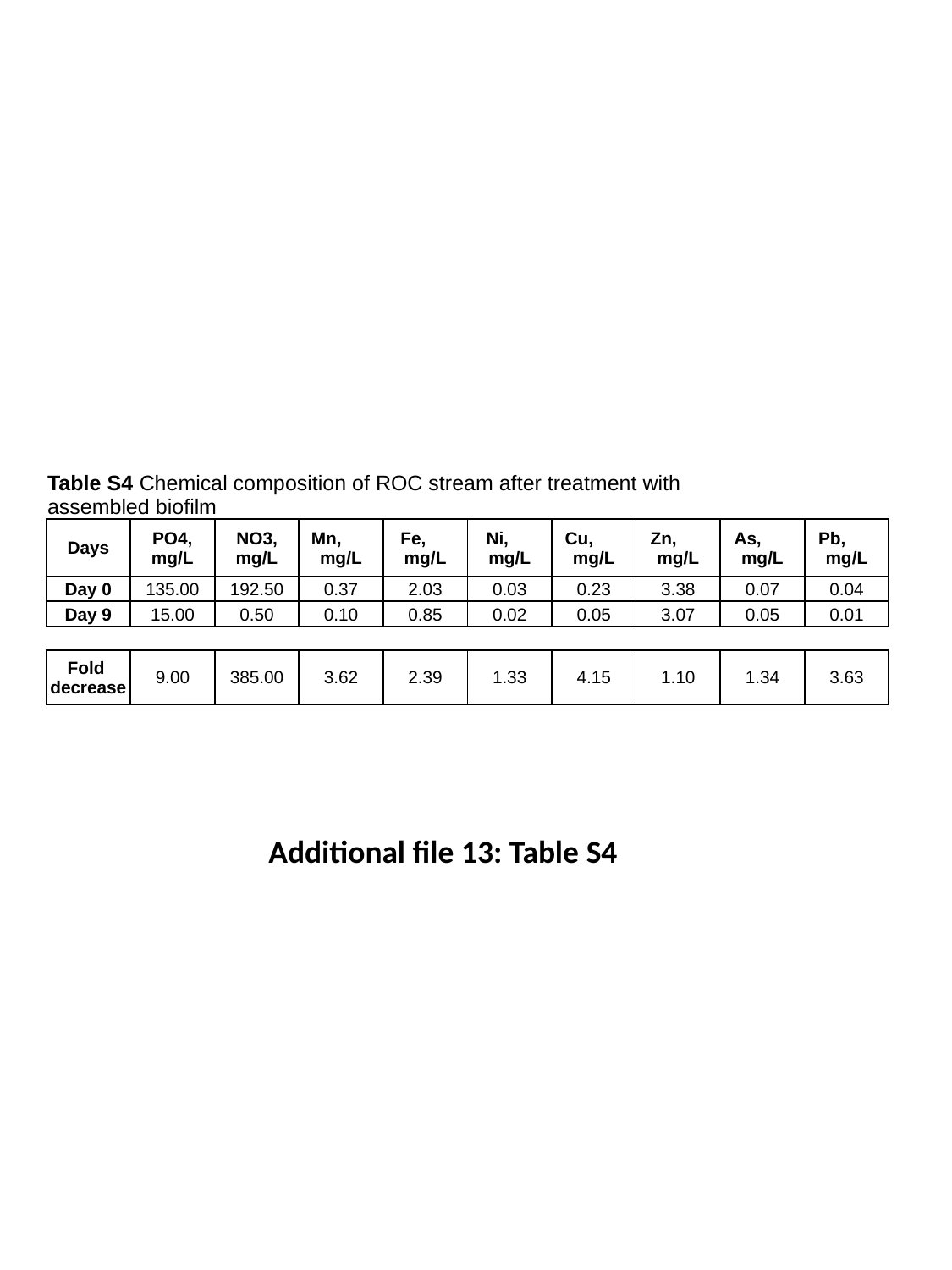

| Table S4 Chemical composition of ROC stream after treatment with assembled biofilm | | | | | | | | | |
| --- | --- | --- | --- | --- | --- | --- | --- | --- | --- |
| Days | PO4, mg/L | NO3, mg/L | Mn, mg/L | Fe, mg/L | Ni, mg/L | Cu, mg/L | Zn, mg/L | As, mg/L | Pb, mg/L |
| Day 0 | 135.00 | 192.50 | 0.37 | 2.03 | 0.03 | 0.23 | 3.38 | 0.07 | 0.04 |
| Day 9 | 15.00 | 0.50 | 0.10 | 0.85 | 0.02 | 0.05 | 3.07 | 0.05 | 0.01 |
| | | | | | | | | | |
| Fold decrease | 9.00 | 385.00 | 3.62 | 2.39 | 1.33 | 4.15 | 1.10 | 1.34 | 3.63 |
Additional file 13: Table S4
