# Supplementary material for: Applications of microalgal biofilms for wastewater treatment and bioenergy production
Source: Biotechnol Biofuels. 2017 May 10;10:120. doi: 10.1186/s13068-017-0798-9 (PMC5424312; doi:10.1186/s13068-017-0798-9)
Supplement: Supplementary file 14 — Additional file 14: Table S5. List of primers used for genotyping of biofilms components. [file 13068_2017_798_MOESM14_ESM.pptx]

## Slide 1
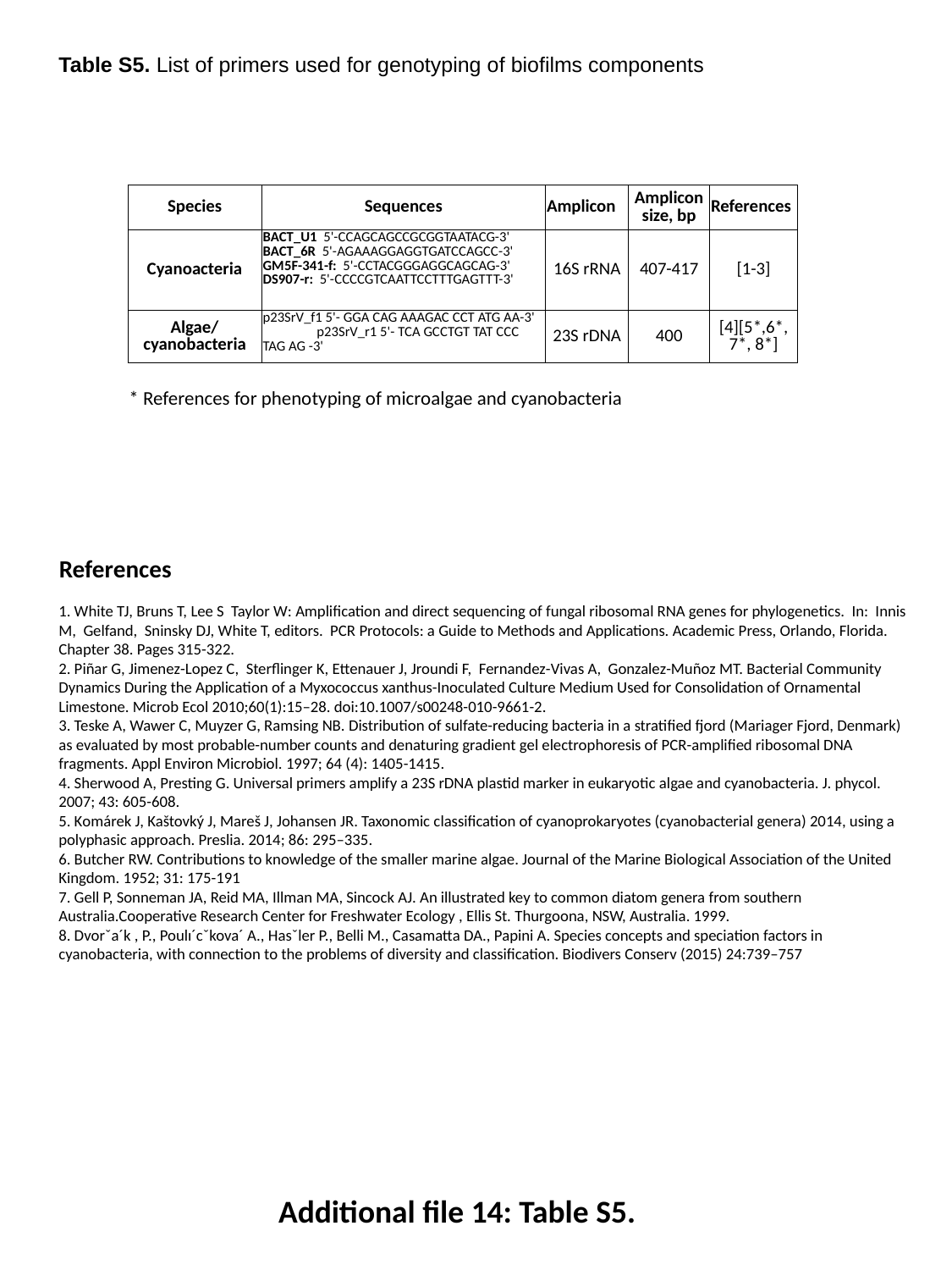

Table S5. List of primers used for genotyping of biofilms components
| Species | Sequences | Amplicon | Amplicon size, bp | References |
| --- | --- | --- | --- | --- |
| Cyanoacteria | BACT\_U1 5'-CCAGCAGCCGCGGTAATACG-3' BACT\_6R 5'-AGAAAGGAGGTGATCCAGCC-3' GM5F-341-f: 5'-CCTACGGGAGGCAGCAG-3' DS907-r: 5'-CCCCGTCAATTCCTTTGAGTTT-3' | 16S rRNA | 407-417 | [1-3] |
| Algae/ cyanobacteria | p23SrV\_f1 5'- GGA CAG AAAGAC CCT ATG AA-3' p23SrV\_r1 5'- TCA GCCTGT TAT CCC TAG AG -3' | 23S rDNA | 400 | [4][5\*,6\*, 7\*, 8\*] |
| | | | | |
| \* References for phenotyping of microalgae and cyanobacteria | | | | |
References
1. White TJ, Bruns T, Lee S Taylor W: Amplification and direct sequencing of fungal ribosomal RNA genes for phylogenetics. In: Innis M, Gelfand, Sninsky DJ, White T, editors. PCR Protocols: a Guide to Methods and Applications. Academic Press, Orlando, Florida. Chapter 38. Pages 315-322.
2. Piñar G, Jimenez-Lopez C, Sterflinger K, Ettenauer J, Jroundi F, Fernandez-Vivas A, Gonzalez-Muñoz MT. Bacterial Community Dynamics During the Application of a Myxococcus xanthus-Inoculated Culture Medium Used for Consolidation of Ornamental Limestone. Microb Ecol 2010;60(1):15–28. doi:10.1007/s00248-010-9661-2.
3. Teske A, Wawer C, Muyzer G, Ramsing NB. Distribution of sulfate-reducing bacteria in a stratified fjord (Mariager Fjord, Denmark) as evaluated by most probable-number counts and denaturing gradient gel electrophoresis of PCR-amplified ribosomal DNA fragments. Appl Environ Microbiol. 1997; 64 (4): 1405-1415.
4. Sherwood A, Presting G. Universal primers amplify a 23S rDNA plastid marker in eukaryotic algae and cyanobacteria. J. phycol. 2007; 43: 605-608.
5. Komárek J, Kaštovký J, Mareš J, Johansen JR. Taxonomic classification of cyanoprokaryotes (cyanobacterial genera) 2014, using a polyphasic approach. Preslia. 2014; 86: 295–335.
6. Butcher RW. Contributions to knowledge of the smaller marine algae. Journal of the Marine Biological Association of the United Kingdom. 1952; 31: 175-191
7. Gell P, Sonneman JA, Reid MA, Illman MA, Sincock AJ. An illustrated key to common diatom genera from southern Australia.Cooperative Research Center for Freshwater Ecology , Ellis St. Thurgoona, NSW, Australia. 1999.
8. Dvorˇa´k , P., Poulı´cˇkova´ A., Hasˇler P., Belli M., Casamatta DA., Papini A. Species concepts and speciation factors in cyanobacteria, with connection to the problems of diversity and classification. Biodivers Conserv (2015) 24:739–757
Additional file 14: Table S5.
